# The accumulation of adverse life events across the life course and their association with mental health and well-being: exploring gender heterogeneity

**DOI:** 10.1186/s12889-026-30076-7

**Published:** 2026-10-23

**Authors:** Simon Hirch

**Affiliations:** Working Group Demography and Health, School of Public Health, Bielefeld University, Bielefeld, Germany

**Keywords:** Adverse life events, Cumulative adversity, Life course, Mental health, Well-being, Gender, Depression, Life satisfaction, Quality of life

## Abstract

**Background:**

Most individuals experience multiple adverse life events across their life course, accumulating into cumulative disadvantage with lasting consequences for mental health and well-being. Evidence suggests that women’s mental health and well-being is more vulnerable to the negative consequences of single adverse life events, due to gender differences in event perception, available resources, and preferred coping strategies. Until now it’s unclear whether these gendered patterns hold when looking at the accumulation of adverse events over a life course. The aim of this study is to examine if the accumulation of adverse life events is associated with depressive symptomatology, life satisfaction, and quality of life with a central focus on gender differences in these relationships.

**Methods:**

A secondary data analysis was conducted using Wave 3 of the English Longitudinal Study of Ageing (ELSA). Accumulation of adverse life events was operationalized as the number of different adverse events experienced during the life course, covering 22 life events. Three accumulation scores were calculated: a total score (TCALE) including all adverse events and two separate scores for self-oriented (SOCALE) and other-oriented (OOCALE) adverse events. Associations with depressive symptomatology, life satisfaction, and quality of life were analyzed using logistic and linear regression models. Interaction terms between accumulation scores and gender were included to examine gender differences in these associations.

**Results:**

Greater accumulation of adverse life events is associated with higher likelihood of depressive symptomatology, lower life satisfaction, and reduced quality of life. Gender moderates the relationships for TCALE, with women being more vulnerable to the accumulation of adverse life events. Although no consistent gender interactions could be found for SOCALE and OOCALE, non-significant trends, for OOCALE, aligned with the patterns observed for the total accumulation score.

**Conclusion:**

Cumulative life course adversity is a significant risk factor for poorer mental health and well-being, with women showing heightened vulnerability.

**Supplementary Information:**

The online version contains supplementary material available at https://doi.org/10.1186/s12889-026-30076-7.

## Introduction

Most individuals experience multiple adverse life events throughout their lives [1, 2], ranging from personal losses and health challenges to major disruptions in social or occupational life. The majority of these events are linked to severe and long-lasting negative consequences for an individual’s mental health and well-being [e.g., 3–10]. Past research primarily focused on individual adverse life events and their impact on mental health and well-being. Although this approach allows the examination of event-specific burden, it risks decontextualizing adverse experiences and overlooking the complex interplay between multiple adversities across the life course [11]. Empirical evidence indicates that adverse life events rarely occur in isolation. They can trigger further adverse events [12–14], interact with each other [15, 16], and accumulate stress over a lifetime [1, 17–19], thereby amplifying their negative consequences for mental health and well-being. This study aims to advance our understanding of how multiple adversities collectively shape mental health and well-being across the life course and how these associations vary by gender.

One of the most common and serious mental health outcomes following an adverse life event is depression [20], which constitutes a major public health challenge [21]. As the most prevalent mental disorder worldwide [22], depression affects about one in ten individuals annually and one in five over a lifetime [23]. Its social consequences are severe, as this mental illness accounts for 5.6% of all years lived with disability [24] and imposes a substantial financial burden on healthcare systems [25].

Beyond depression, adverse life events have also been shown to be associated with poorer well-being outcomes, including life satisfaction and quality of life [26, 27]. These two measures of well-being are key markers of societal health [27, 28] as they are closely linked to various mental [27, 29–32] and physical health outcomes [28, 29, 33–35]. Because of their immense relevance for both individual and population health, measures of life satisfaction and quality of life are increasingly included in economic and political decision-making [36, 37].

Although depressive symptomatology, life satisfaction, and quality of life are related constructs of mental health and well-being, they capture distinct dimensions of individuals’ experiences. Depressive symptomatology primarily reflects affective and emotional distress [38], life satisfaction represents a person’s evaluation of his or her own life in relation to one’s own expectations and standards [39], and quality of life encompasses a broader assessment of an individual’s life and living conditions across different domains, including physical and social functioning, material and emotional well-being, and personal development (26). Examining all three outcomes therefore provides a comprehensive picture of the consequences of accumulated adverse life events for mental health and well-being.

Given their importance for individual and societal health, it is crucial for the public health sector to understand how depression, life satisfaction, and quality of life are shaped by the accumulation of adverse life events over the life course in order to design interventions that mitigate long-term mental health and well-being inequalities associated with cumulative adversity.

Although there is no universal definition of what characterizes an adverse life event, prior literature highlights several key characteristics of such events. Adverse life events are events that have the potential to significantly alter a person’s social world [40], require considerable adjustment in daily life or coping strategies [41], and are perceived as a threat to one’s own or close others’ life, physical, or mental well-being [42, 43]. Examples of adverse life events include the death of a loved one, loss of a home due to a natural disaster, or a divorce. It should be noted that some adverse events, such as being the victim of sexual assault, are largely exogenous, while others, for example, widowhood, are often accompanied by chronic stressors that precede the event, such as spousal caregiving, or follow it, such as financial strain. Adverse life events that involve chronic stressors have been shown to be particularly burdensome in the context of accumulated adversities, as chronic stress is not only associated with poorer mental health and well-being but also amplifies the burden associated with subsequent stressors [44].

Understanding the characteristics of adverse life events provides a foundation for examining how burdens caused by these events accumulate over a lifetime. In this study, accumulation is understood as the progressive aggregation of adverse life events during a person’s life. Life-course theory states that adversities are embedded in social and historical contexts and that earlier experiences as well as critical transitions shape vulnerability to subsequent stressors [43, 45, 46]. Building on this perspective, stress-proliferation and cumulative-inequality theory together describe how initial adversities can generate further stressors, which additionally negatively affect a person’s mental health and well-being, as well as socioeconomic and social resources, that are necessary to cope with further adverse events. This results in a self-reinforcing process of increasing disadvantage [16, 47], in which the accumulation of adverse life events is a dynamic process where adversities generate additional stressors and resource losses that interact with each other and shape long-term mental health and well-being.

Building on this accumulation and self-reinforcing process, studies have shown that the accumulation of adverse life events is associated with a higher probability of clinical depression or depressive symptomatology [1, 2, 18, 48–58], lower life satisfaction [1, 2, 6, 18, 53, 59], and reduced quality of life [18]. Despite broad empirical consensus, two studies examining older Israeli adults report divergent findings, observing no association between the accumulation of adverse life events and quality of life [60] and an association with depressive symptomatology only for one of two depression indicators examined [55]. One possible explanation for these contrasting findings is the exceptionally high lifetime exposure to collective stressors and trauma among older Israeli adults, including the Holocaust, large-scale migration, and repeated military conflicts [61, 62], which may shape how adverse events are experienced and processed [55]. Notably, the specific adverse events considered differ substantially across studies, reflecting variations in data sources and event selection.

Prior research on the consequences of the accumulation of adverse life events for mental health and well-being highlights the importance of distinguishing events based on their primary orientation into self- and other-oriented adverse life events [18, 55, 56, 60, 63]. This distinction is grounded in a change in the definition of traumatic events in the Diagnostic And Statistical Manual Of Mental Disorders (DSM-IV), published in 1994, which extended the concept of traumatic events to include events affecting close others [42], thereby acknowledging the interconnectedness of individuals’ lives and the ways in which experiences of close others can shape one’s own life course, mental health, and well-being [45]. Self-oriented adverse life events directly affect the individual (e.g., serious illness, divorce, or sexual assault), whereas other-oriented adverse life events primarily impact close others (e.g., death of a loved one, witnessing a fatal accident, or parental divorce). This distinction of adverse events, however, does not solely depend on who is primarily affected by the event but also on the coping role adopted. Other-oriented adverse events often place individuals in caregiving, supporting, or grieving roles, whereas self-oriented events mainly require individual emotion regulation and self-focused coping [55]. Prior studies suggest that the accumulation of self-oriented adverse life events leads to more intense and direct psychological distress than the accumulation of other-oriented adverse events [18, 55, 56, 60, 63]. This difference may be explained by other-oriented events often coming with more meaningful role-taking [64, 65], greater fostering of social interactions [66], and larger emotional distance to the adverse consequences [67], all together buffering their negative impact on mental health and well-being compared with self-oriented adverse events.

Across studies, the accumulation of self-oriented adverse events is consistently linked to a higher risk of depression [18, 55, 56, 60] as well as lower life satisfaction [18, 56] and quality of life [18, 56]. In contrast, evidence for the accumulation of other-oriented adverse events is less clear and somewhat conflicting. Studies in older Israeli populations showed mixed findings: one study found an association between accumulated other-oriented events and increased depressive symptoms but not lifetime depression diagnoses, alongside higher quality of life [60]; another study found no or even a negative association with depressive symptoms depending on the measurement instrument used [19]. When accounting for life stage, analyses in an Israeli population showed that other-oriented adverse events during childhood were linked to fewer depressive symptoms as well as higher life satisfaction and quality of life, while such events during adulthood showed largely non-significant associations with depression and well-being [56]. In contrast, evidence from an older English population linked accumulated other-oriented events to higher risk of experiencing depression as well as lower life satisfaction and decreased quality of life, regardless of the life stage the adversities occurred [18]. Overall, while the accumulation of self-oriented adverse events reliably predicts poorer mental health and reduced well-being, findings on accumulated other-oriented events remain heterogeneous and vary across context and outcome measures, highlighting the need for further research.

A large body of research suggests that the negative consequences of adverse life events on mental health and well-being are highly gendered, with women experiencing a greater burden than men for most events [20, 68–70].

Women consistently report perceiving adverse life events as more stressful, less controllable, and more disruptive than men, particularly when these events involve close others [69, 71, 72]. This heightened impact of other-oriented adversity could be explained by women’s stronger relational orientation [73, 74] and their greater tendency to define themselves through their close interpersonal relationships [75, 76], compared to men. As a result, adverse events affecting loved ones are particularly striking for women and may contribute more strongly to cumulative burden and long-term negative consequences for mental health and well-being.

Building on gendered patterns in the perception of adverse life events, differences in coping resources and strategies, which are crucial for managing adverse events, may further shape gendered consequences of accumulated adversity for mental health and well-being. Studies report that men tend to possess greater personal resources, such as internal locus of control, self-efficacy, or optimism [77–82], as well as material resources like income and wealth [83, 84]. Women, in contrast, typically maintain larger and more supportive social networks [85, 86]. Nevertheless, it has been shown that men are less reliant on their social network to cope with adverse life events than women due to the other resources available to them [87–89]. In addition to gender differences in available coping resources, gender also shapes preferred coping strategies. It has been shown that women tend to rely on emotion-oriented coping strategies such as venting, rumination, or seeking emotional support, while men are more likely to use problem-focused strategies, including planning and active problem solving [82, 90–93]. This is of great importance, since problem-focused strategies have been shown to offer greater protection against the long-term negative consequences of adverse life events on mental health and well-being [94, 95].

These individual resources and coping strategies are embedded in the structural conditions that individuals experience over the life course. Women, particularly in older generations, have historically had less access to educational opportunities [96, 97], more fragmented employment trajectories [97], and greater caregiving responsibilities [98] than men. Such structural constraints may further contribute to gender differences in the mental health and well-being consequences of adverse life events [43, 99].

Overall, these gender differences in perception, available resources, preferred coping strategies, and exposure to structural constraints provide a theoretical basis for expecting gender-specific effects in the relationship between the accumulation of adverse life events and depressive symptomatology, life satisfaction, and quality of life, with women being particularly vulnerable. This may be especially pronounced for the accumulation of other-oriented adverse events, which women tend to perceive as more stressful and disruptive than men, potentially intensifying gendered consequences.

Previous research on gender differences in the association between the accumulation of adverse life events over the life course with mental health and well-being is still limited, with only one study reporting greater vulnerability for women in terms of depressive symptomatology [52]. No study so far examined gender differences in the relationship between accumulated adverse life events and measures of well-being. In addition, the potential role of event orientation in shaping gendered effects has not yet been considered.

Building on the theoretical considerations and empirical findings presented above, this study examines how the accumulation of adverse life events across the life course is related to depressive symptomatology, life satisfaction, and quality of life, with a special focus on gender differences and event orientation. It is expected that the accumulation of adverse life events overall as well as separately for self- and other-oriented adverse events is associated with a greater likelihood of depressive symptomatology, lower life satisfaction, and reduced quality of life. In addition, gender is expected to moderate these relationships, with women being more vulnerable to the negative consequences of accumulated adversity across overall, self-oriented, and other-oriented life events.

This study contributes to the literature on the accumulation of adverse life events and their consequences on mental health and well-being in several ways. First, by examining a broad range of adverse events, it provides a complementary perspective, strengthening existing evidence on the association between cumulative exposure to adversity and mental health and well-being. Second, it clarifies the role of event orientation, particularly shedding light on the accumulation of other-oriented events, for which previous findings have been mixed. Third, it provides additional evidence for the role of gender in the association between cumulative adversity and depression and offers novel insights into gendered associations in the relationship between cumulative adversity and life satisfaction as well as quality of life. Finally, for the first time, this study examines how event orientation interacts with gender in the accumulation of adverse life events, providing a more nuanced understanding of how mental health and well-being are shaped by their combined effects.

Taken together, this study aims to advance our understanding of how the cumulative burden caused by adverse life events shapes men’s and women’s mental health and well-being. This can help to identify high-risk populations and inform the development of interventions and preventive strategies that take into account not only single adverse life events but also the broader life-course context in which the burdens of adverse experiences can accumulate.

## Methods

### Study sample

The current study utilizes data from the English Longitudinal Study of Ageing (ELSA), a nationally representative study of older adults aged 50 + and their partners living in private households in England [100, 101]. The analyses were restricted to data from Wave 3, conducted in 2006–2007, as this is the only wave including a life history interview together with a self-completion questionnaire, both of which were used to collect detailed retrospective information on a wide range of life events throughout an individual’s life course [102].

Within the life history interview, life events were recorded using a Life History Calendar, an interview-based method for collecting information about past life events. It has the form of a calendar that depicts different areas of life (e.g., living, work, family) over a timeline and includes socially important events. By entering personal events alongside predefined socially meaningful events, it is easier for respondents to recall and chronologically place events. This method improves the accuracy and completeness of retrospective data on life events. Whereas the life history interview was conducted face-to-face, the self-completion questionnaire was filled out alone on paper, giving respondents more time to think as well as greater privacy [102].

A total of 9,771 individuals participated in the ELSA survey in Wave 3. Only participants who completed both the life history interview and the self-completion questionnaire were retained in the sample (excluded = 3,189). Next, observations with missing information on any of the three outcomes (missing values = 956), at least one adverse life event (missing values = 547), or at least one confounder (missing values = 15) were stepwise removed. This results in a final analytical sample comprising 5,064 individuals.

As approximately 48% of the initial sample (*n* = 4,707 observations) were excluded from the analytical sample due to missing information, comparisons between included and excluded individuals were conducted across key characteristics used in subsequent analyses to assess potential selection bias. Results of this comparison are presented in Additional File 1.

### Outcomes

Depressive symptomatology was assessed using the eight-item Center for Epidemiological Studies Depression Scale (CES-D), a widely used screening test for assessing depression based on the number of depressive symptoms [100, 103]. It should be noted that this measure does not directly assess clinical depression, as depressive symptomatology may occur without meeting diagnostic criteria [21]. Participants were asked to indicate (yes/no) whether they had experienced each of eight depressive symptoms for much of the time during the last week: (1) feeling depressed, (2) feeling that everything was an effort, (3) having restless sleep, (4) feeling lonely, (5) feeling sad, (6) being unable to get going, (7) not enjoying life, and (8) not feeling happy. Two items were initially positively worded (“enjoyed life” and “felt happy”) and, in the following, reverse-coded to ensure that all items were scaled in the same direction. Responses were then summed to create a total score ranging from 0 to 8. Depressive symptomatology was defined as the presence of four or more symptoms of depression, consistent with previous studies [104, 105]. This cut-off point has been found to produce comparable results to the ≥ 16 cut-off point for the well-validated 20-item CES-D scale [103, 106].

Life satisfaction was assessed using the Satisfaction with Life Scale [39], a commonly used five-item scale designed to measure life satisfaction. The scale consists of the following items: (1) “In most ways my life is close to my ideal”, (2) “The conditions of my life are excellent”, (3) “I am satisfied with my life”, (4) “So far I have got the important things I want in life”, and (5) “If I could live my life again, I would change almost nothing”. Responses were originally recorded on a 7-point Likert scale ranging from 1 (“strongly agree”) to 7 (“strongly disagree”). To facilitate interpretation, all item responses were shifted by subtracting one point, resulting in a scale ranging from 0 to (6). Item scores were summed to generate a total life satisfaction score, which was subsequently reverse-coded so that higher values indicated greater life satisfaction. The final score ranged from 0 to 30.

To assess quality of life, the Control, Autonomy, Self-realization, and Pleasure 19 scale (CASP-19 scale), a validated instrument for measuring quality of life in older adults, was used [107–109]. The CASP-19 scale consists of nineteen items across four different domains, which are shown in Table 1. The questions begin with the phrase, “Here is a list of statements that people have used to describe their lives or how they feel. How often do you feel like this?”. Responses were recorded on a four-point Likert scale ranging from 0 (“never”) to 3 (“often”). Six items (1, 2, 3, 6, 8, and 9) were negatively worded and therefore reverse-coded so that higher values consistently indicated better quality of life. Item responses were summed to create a total CASP-19 score ranging from 0 to 57, with higher scores reflecting higher quality of life [109].

**Table 1:** Domains and items in the CASP-19

Domain	Items
Control	1. My age prevents me from doing the things I would like to do^a^
	2. I feel that what happens to me is out of my control^a^
	3. I feel free to plan for the future
	4. I feel left out of things^a^
Autonomy	5. I can do the things I want to do
	6. Family responsibilities prevent me from doing the things I want to do^a^
	7. I feel that I can please myself what I do
	8. My health stops me from doing the things I want to do^a^
	9. Shortage of money stops me from doing things I want to do^a^
Pleasure	10. I look forward to each day
	11. I feel that my life has meaning
	12. I enjoy the things that I do
	13. I enjoy being in the company of others
	14. On balance, I look back on my life with a sense of happiness
Self-realization	15. I feel full of energy these days
	16. I choose to do things that I have never done before
	17. I feel satisfied with the way my life has turned out
	18. I feel that life is full of opportunities
	19. I feel that the future looks good for me

^a^ Negatively worded and for the analysis reverse coded

### Accumulation of adverse life events

Selection of adverse life events was guided by the Cumulative Lifetime Adversity Measure (CLAM) [59], a frequently used instrument for capturing the accumulation of adverse life events across the lifespan [1, 59, 110, 111]. This measure is based on the “trauma section” of the Diagnostic Interview Schedule [112] and has been expanded to include additional stressful life events identified from primary care patient reports [113].

Although the CLAM already covers a broad spectrum of adverse life events, this list is still not exhaustive and therefore must be supplemented with selected additional events [110]. Subsequently, two further adverse events available in ELSA, first unexpected job loss and second separation from a long-term or cohabiting partner, were additionally included. This results in a total of 22 different adverse life events considered in the analyses. For three of these, only experiences up to age 16 were recorded.

As information on the frequency of occurrence for individual adverse life events was not available for most events, each event was coded as a dichotomous variable (0 = never experienced, 1 = ever experienced). Cumulative adverse life event exposure was therefore operationalized as the total number of different adverse life events experienced throughout an individual’s life course, thereby assuming that each type of adverse life event contributes equally to the cumulative exposure. This approach is widely used in prior research [1, 114] and resulted in a total cumulative adverse life event score (TCALE), which increases by 1 for every adverse life event experienced, ranging from 0 to 22.

Adverse life events were further categorized as self- or other-oriented following prior definitions and previous literature [18, 55, 56]. Of the 22 adverse life events considered, 12 were classified as self-oriented and 10 as other-oriented. This resulted in a self-oriented cumulative adverse life event score (SOCALE), ranging from 0 to 12, and an other-oriented cumulative adverse life event score (OOCALE), ranging from 0 to 10. All adverse life events included in the analyses, together with their orientation, are presented in Table 2.

**Table 2:** Descriptive distribution of experienced adverse life events and accumulation scores, stratified by gender

	Men (*n* = 2232)	Women (*n* = 2832)	*p*-value^b^
Self-oriented adverse life events^a^			
Divorce	458 (20.5)	653 (23.1)	0.033
Separation from a long-term or/and cohabiting partner	397 (17.8)	612 (21.6)	0.001
Diagnosis of a serious physical disease	1248 (55.9)	1716 (60.6)	0.001
Job loss due to company closing	585 (26.2)	523 (18.5)	< 0.001
Experienced a natural disaster	289 (12.9)	249 (8.8)	< 0.001
Suffered a life-threatening illness or serious accident	684 (30.6)	618 (21.8)	< 0.001
Victim of a serious physical attack or assault	152 (6.8)	127 (4.5)	< 0.001
Victim of sexual assault	62 (2.8)	243 (8.6)	< 0.001
Fired a weapon or came under fire during combat	245 (11.0)	16 (0.6)	< 0.001
Experienced severe financial hardship	391 (17.5)	577 (20.4)	0.011
Physically abused by parents before age 16	67 (3.0)	99 (3.5)	0.368
Experienced evacuation during war	249 (11.2)	234 (8.3)	0.001
Other-oriented adverse life events^a^			
Death of long-term partner/spouse	223 (10.0)	599 (21.2)	< 0.001
Death of child	118 (5.3)	181 (6.4)	0.111
Death of father	261 (11.7)	322 (11.4)	0.754
Death of mother	250 (11.2)	237 (8.4)	0.001
Parental divorce before age 16	108 (4.8)	148 (5.2)	0.576
Experienced violence between parents before age 16	382 (17.1)	619 (21.9)	< 0.001
Witnessed serious injury or death in war or military action	234 (10.5)	37 (1.3)	< 0.001
Witnessed a fatal or violent accident (not war)	487 (21.8)	206 (7.3)	< 0.001
Lost a very close friend or relative in war or military service	285 (12.8)	223 (7.9)	< 0.001
Close friend or relative was at risk of death or died due to illness or serious accident	407 (63.0)	1871 (66.1)	0.027
Accumulation scores			
TCALE	3.84 (2.21)	3.57 (1.99)	< 0.001
SOCALE	2.16 (1.50)	2.00 (1.48)	< 0.001
OOCALE	1.68 (1.27)	1.57 (1.09)	0.001

Absolute numbers (percentages) are presented for individual adverse life events; mean values (standard deviation) are presented for accumulation scores

^a^ For single adverse life events, only participants who reported experiencing the event are shown. Non-experienced cases (coded as “never experienced”) are not presented

^b^ p-values indicating gender differences from chi-square tests for single adverse life events or two-tailed t-tests for accumulation scores

*Abbreviations*: *TCALE *total cumulative adverse life event score, *SOCALE*  self-oriented cumulative adverse life event score, *OOCALE*  other-oriented cumulative adverse life event score

### Covariates

To account for potential confounders in the association between the accumulation of adverse life events and mental health and well-being, several control variables were considered.

Selecting confounders in the context of the accumulation of adverse life events is challenging, as individual characteristics can serve multiple roles in the theoretical causal pathway. Two issues are of particular relevance. First, some variables may function both as confounders and as part of the exposure itself. For example, marital status is an important confounder when examining associations between adverse events such as unexpected job loss or a life-threatening illness and mental health and well-being [115, 116]. At the same time, marital status also includes the adverse life events of divorce or widowhood, which are considered in the accumulation scores and are independently associated with mental health and well-being [117–119]. Adjusting for such variables may therefore control for parts of the exposure, potentially leading to an underestimation of the effects.

Second, some variables may act as both confounders and mediators. Income, for instance, is a well-established confounder in the relationship between many adverse life events and mental health/well-being outcomes [57, 120]. At the same time, it is also heavily shaped by the accumulation of adverse events [121, 122]. In the ELSA data, income (as well as all other covariates) is surveyed at the time of the interview and therefore after the adverse events were experienced, making it primarily a product of cumulative adversity. Adjusting for such variables could block mediation pathways, resulting in an underestimation of the association.

Given these considerations, it was decided to adjust only for stable confounders that are relevant across all adverse life events and closely linked to the outcomes. These include gender (men vs. women), age (≤ 59, 60–69, 70–79, 80+), and place of birth (UK vs. outside of UK). In addition to these sociodemographic characteristics, the highest level of education attained was also considered, as education is typically established early in life and remains relatively stable in adulthood [123, 124]. Education was categorized into four levels (no qualification, low education, medium education, high education). The “no qualification” category includes individuals without a formal school leaving certificate. “Low education” covers basic schooling or foreign qualifications that cannot be clearly classified. “Medium education” includes secondary/high school diplomas, and “high education” comprises university or other post-secondary degrees.

### Statistical analysis

Descriptive statistics of adverse life events were presented separately for men and women, with absolute frequencies and percentages reported for each individual event and mean values with standard deviations calculated for the three accumulation scores. Gender differences were tested using Pearson chi-square tests for individual events and two-tailed t-tests for the accumulation scores. Overall sample characteristics were summarized for the total sample and by gender, with frequencies and percentages for nominal and ordinal variables and means with standard deviations for continuous variables.

Regression models were used to examine the association between the three accumulation scores and the considered outcomes. Logistic regression models were applied for the dichotomous outcome depressive symptomatology, whereas linear regression models were used for the metric outcome’s life satisfaction and quality of life. All models were estimated with standard errors clustered at the household level to account for the potential dependence between respondents from the same household.

Some previous research suggests a potential quadratic association between the accumulation of adverse life events and mental health and well-being [54, 59, 110]. To examine this hypothesis, quadratic terms of the three accumulation scores were included in the regression models for all outcomes. Smoothing splines were additionally fitted for the three accumulation scores as a further check for non-linearity. As the quadratic terms were not statistically significant and the smoothing splines showed no substantial deviations from the linear pattern (with deviations only occurring at the upper end of the distribution where 10 or fewer observations were available) in any model (see Additional File 2), the linear terms were retained.

For each outcome, four regression models were estimated on the full sample, all adjusted for the confounders described above. Model 1 included TCALE, whereas Model 2 included SOCALE and OOCALE. To examine potential moderation effects of gender, Model 3 extended Model 1 and Model 4 extended Model 2 by adding interaction terms between the respective adverse life event score and gender.

To visualize the moderating effects of gender, marginal predicted probabilities (for depressive symptomatology) and marginal means (for life satisfaction and quality of life) were calculated and averaged across the observed distribution of all covariates. Figures display 84% confidence intervals to facilitate visual group comparisons, as non-overlapping 84% confidence intervals between two groups correspond approximately to a statistically significant difference at the 5% level [125, 126]. Based on the results of the interaction analyses and theoretical considerations, additional gender-stratified regression models were estimated, which are presented in Additional File 3.

A significance level α of 0.05 was applied to all statistical tests. The statistical analysis was performed using R version 4.5.1 [127].

### Sensitivity analyses

To check the robustness of the results against modifications of the constructs and procedures used, four sensitivity analyses were conducted.

First, an alternative operationalization to the checklist-based measurement used to capture the accumulation of adverse life events was examined. In the main analysis, the accumulation scores were based on the number of different adverse events ever experienced, as 16 of the available 22 adverse life events were surveyed only in dichotomous form. For 6 events (divorce, death of a long-term partner/spouse, death of a child, separation from a long-term or cohabiting partner, diagnosis of a serious physical disease, and job loss due to company closure), information on frequency of occurrence across the life course was available. Because the repeated exposure to the same adverse event still negatively affects mental health and well-being [128–131], these six adverse life events were not dichotomized in this sensitivity analysis. Instead, the accumulation scores incorporated the number of times each of these adverse events occurred, whereas the other 16 events remained dichotomous. This allowed the accumulation scores to increase with each additional occurrence of the 6 events, making them theoretically open-ended.

The second sensitivity analysis addresses a raised criticism of using an unweighted sum score to measure the accumulation of adverse life events. Critics have argued that an unweighted sum score does not account for the different severity or impact of individual adverse life events [132]. To address this, general weights for each adverse event were assigned, derived from a study in which participants were asked to rate life events on a 0–100 scale according to their perceived stress and adjustment demands [133]. Using these weights, the accumulation of adverse life events is now conceptualized as the sum of the total stress experienced through adverse life events over the life course. Although these weights do not capture the individual-level perceived burden of the events, they provide an alternative perspective on the examined associations, accounting for the type and intensity of the events. The assigned weights are shown in Additional File 4.

Third, robustness to the inclusion of additional confounders into the regression models was tested. These include current employment status (in labor force, unemployed, retired), equivalized household income (calculated by dividing the household income of one individual by the square root of the number of household members) divided into tertiles (low, medium, high), partnership status (married, cohabiting, neither), number of children under the age of 18 living in the household, social participation, and social network size. Equivalized household income tertile cut-offs were determined using the full sample prior to excluding observations with missing values. Social participation was measured by the number of memberships (yes/no) in eight types of organizations, clubs, and societies, resulting in a sum score ranging from 0 to 8 [134, 135]. Social network size was calculated as the sum of close relationships with children, other family members, and friends, with each category top-coded at 10 to limit the influence of outliers, resulting in a total score from 0 to 30 [134, 136, 137]. All confounders were measured at Wave 3, with no information available on these characteristics at the time the adverse life events occurred. Individuals with missing data on the additional confounders were excluded, resulting in a sample of 4,183 instead of 5,064 participants for this sensitivity analysis.

The fourth sensitivity analysis considers alternative operationalizations of the examined outcomes. For depressive symptomatology, a more sensitive cut-off of ≥ 3 symptoms (instead of ≥ 4) was used, as in some other previous ELSA studies [18, 138]. Quality of life was measured in this sensitivity analysis using the CASP-12, a shortened version of the CASP-19 scale [139], which includes items 1, 2, 4, 5, 7, 9, 10, 11, 12, 15, 18, and 19, shown in Table 1 [108], resulting in a slightly modified operationalization of quality of life.

Overall, the main results were largely robust to variations in measurement, covariate inclusion, and outcome definitions. Some interaction terms between the accumulation scores and gender that were significant in the main analysis marginally lost significance in supplementary analyses, likely due to reduced statistical power, partial adjustment for exposure pathways, or increased measurement complexity. Yet the observed trends in gender differences remained consistent. Full descriptive statistics and regression results of the sensitivity analyses are presented in Additional File 5.

## Results

### Descriptive results

Table 3 shows the general characteristics of the study population stratified by gender and for the entire sample. The sample consists of 2232 men and 2832 women, with women making up the larger proportion of the study population (55.9%). Depressive symptomatology was observed in 8.8% of men and 13.9% of women. Mean life satisfaction scores were 20.37 among men and 20.04 among women, whereas the average quality of life score was 41.49 among men and 41.83 among women. Additional descriptive information on the life satisfaction score and the quality of life score, comprising their 25th percentile, median, and 75th percentile, is shown in Additional File 6.

**Table 3:** General characteristics of the study population stratified by gender and overall

	Men (*n* = 2232)	Women (*n* = 2832)	Overall (*n* = 5064)	*p*-value^a^
Depressive symptomatology				< 0.001
No	2035 (91.2)	2438 (86.1)	4473 (88.3)	
Yes	197 (8.8)	394 (13.9)	591 (11.7)	
Life satisfaction score				0.069
Mean (SD)	20.4 (6.3)	20.04 (6.47)	20.18 (6.4)	
Quality of life score (CASP-19)				0.152
Mean (SD)	41.5 (8.4)	41.8 (8.5)	41.68 (8.4)	
Age				0.013
≤ 59 years	754 (33.8)	1079 (38.1)	1833 (36.2)	
60–69 years	740 (33.2)	866 (30.6)	1606 (31.7)	
70–79 years	544 (24.4)	640 (22.6)	1184 (23.4)	
80 + years	194 (8.7)	247 (8.7)	441 (8.7)	
Birthplace				0.373
UK	2115 (94.8)	2666 (94.1)	4781 (94.4)	
Outside of UK	117 (5.2)	166 (5.9)	283 (5.6)	
Highest education				< 0.001
No qualification	424 (19.0)	761 (26.9)	1185 (23.4)	
Low education	602 (27.0)	1013 (35.8)	1615 (31.9)	
Middle education	641 (28.7)	602 (21.3)	1243 (24.5)	
High education	565 (25.3)	456 (16.1)	1021 (20.2)	

Absolute numbers (percentages) are presented for nominal and ordinal variables; mean values (standard deviation) are presented for continuous variables

^a^ *p*-values indicating gender differences from chi-square tests for categorical variables or two-tailed t-tests for continuous characteristics

*Abbreviations*: *SD*  standard deviation, *CASP-19*  Control, Autonomy, Self-realization, and Pleasure 19, *UK*  United Kingdom

Table 2 shows the descriptive distribution of individual adverse life events experienced, as well as the three accumulation scores, stratified by gender, along with the test results for gender differences. It can be seen that women reported more frequently that they experienced divorce (23.1% vs. 20.5% for men; *p* = 0.033), separation from a long-term and/or cohabiting partner (21.6% vs. 17.8% for men; *p* = 0.001), diagnosis of a serious physical disease (60.6% vs. 55.9% for men; *p* = 0.001), sexual assault (8.6% vs. 2.8% for men; *p* < 0.001), severe financial hardship (20.4% vs. 17.5% for men; *p* = 0.011), death of a long-term partner/spouse (21.2% vs. 10% in men; *p* < 0.001), violence between parents before age 16 (21.9% vs. 17.1% in men; *p* < 0.001), and that a close friend or relative was at risk of death or dying (66.1% vs. 63.0% in men; *p* = 0.027).

In contrast, men reported higher prevalence of job loss due to company closure (26.2% vs. 18.5% for women; *p* < 0.001), exposure to natural disasters (12.9% vs. 8.8% for women; *p* < 0.001), life-threatening illness or serious accident (30.6% vs. 21.8% for women; *p* < 0.001), being the victim of a serious physical attack or assault (6.8% vs. 4.5% for women; *p* < 0.001), having used a weapon or having combat experience (11.0% vs. 0.6% for women; *p* < 0.001), having to leave their home because of a war (11.2% vs. 8.3% for women; *p* = 0.001), experiencing the death of one’s mother (11.2% vs. 8.4% for women; *p* = 0.001), witnessing a serious injury or death in war or military action (10.5% vs. 1.3% for women; *p* < 0.001), witnessing a fatal or violent accident outside of war (21.8% vs. 7.3% for women; *p* < 0.001), and the loss of a very close friend or relative in war or military service (12.8% vs. 7.9% for women; *p* < 0.001).

No significant gender differences were observed in the lifetime experience of physical abuse by parents (3.0% for men vs. 3.5% for women; *p* = 0.368), parental divorce (4.8% for men vs. 5.2% for women; *p* = 0.576), the death of a child (5.3% for men vs. 6.4% for women; *p* = 0.111), and experiencing the death of one’s own father (11.7% of men vs. 11.4% of women; *p* = 0.754).

Descriptive analysis of the accumulation scores shows that men reported a higher total number of adverse life events than women (3.84 for men vs. 3.57 for women; *p* < 0.001), with this pattern remaining consistent when looking at self-oriented (2.16 for men vs. 2.00 for women; *p* < 0.001) and other-oriented adverse life events (1.68 for men vs. 1.57 for women; *p* = 0.001) separately.

Zero-order correlations between the three accumulation scores were calculated, showing correlations of *r* = 0.84 between TCALE and SOCALE, *r* = 0.72 between TCALE and OOCALE, and *r* = 0.23 between SOCALE and OOCALE. Additional descriptive information on the accumulation scores, comprising their 25th percentile, median, and 75th percentile, is provided in Additional File 6.

### Regression results

Table 4 presents the results of the logistic regression models examining the association between the accumulation scores TCALE, SOCALE, and OOCALE with depressive symptomatology. Each additional adverse life event is associated with 30% higher odds of experiencing depressive symptomatology (OR = 1.30, 95% CI: [1.25; 1.35]; Model 1). When distinguishing by event orientation, each additional self-oriented event is associated with 37% higher odds of experiencing depressive symptomatology (OR = 1.37, 95% CI: [1.29; 1.45]; Model 2), while each additional other-oriented event is associated with 19% higher odds (OR = 1.19, 95% CI: [1.11; 1.29]; Model 2).

**Table 4:** Logistic regression models predicting depressive symptomatology

	Model 1	Model 2	Model 3	Model 4
TCALE	1.30 *** [1.25; 1.35]	-	1.24 *** [1.16; 1.31]	-
SOCALE	-	1.37 *** [1.29; 1.45]	-	1.30 *** [1.18; 1.43]
OOCALE	-	1.19 *** [1.11; 1.29]	-	1.16 * [1.03; 1.29]
TCALE x Gender	-	-	1.09 * [1.00; 1.18]	-
SOCALE x Gender	-	-	-	1.08 [0.96; 1.22]
OOCALE x Gender	-	-	-	1.07 [0.92; 1.24]
Gender: Men (ref.)	-	-	-	-
Women	1.68 *** [1.39; 2.03]	1.67 *** [1.38; 2.02]	1.15 [0.76; 1.74]	1.19 [0.79; 1.81]
Age: ≤ 59 years (ref.)	-	-	-	-
60–69 years	0.87 [0.69; 1.08]	0.83 [0.66; 1.04]	0.87 [0.69; 1.08]	0.83 [0.66; 1.05]
70–79 years	0.75 * [0.59; 0.95]	0.73 * [0.57; 0.93]	0.75 * [0.59; 0.96]	0.73 * [0.57; 0.94]
80 + years	0.90 [0.65; 1.26]	0.93 [0.67; 1.29]	0.91 [0.66; 1.27]	0.94 [0.67; 1.30]
Birthplace: UK (ref.)	-	-	-	-
Outside of UK	1.60 ** [1.13; 2.28]	1.65 ** [1.16; 2.35]	1.62 ** [1.14; 2.30]	1.66 ** [1.17; 2.36]
Highest education: No qualification (ref.)	-	-	-	-
Low education	0.60 *** [0.48; 0.74]	0.60 *** [0.48; 0.75]	0.60 *** [0.48; 0.74]	0.60 *** [0.48; 0.75]
Medium education	0.48 *** [0.37; 0.62]	0.48 *** [0.38; 0.62]	0.48 *** [0.37; 0.62]	0.48 *** [0.37; 0.62]
High education	0.27 *** [0.20; 0.37]	0.27 *** [0.20; 0.37]	0.27 *** [0.19; 0.37]	0.27 *** [0.19; 0.37]
Intercept	0.06 *** [0.04; 0.09]	0.06 *** [0.05; 0.09]	0.08 *** [0.05; 0.12]	0.08 *** [0.05; 0.12]

Shown values are odds ratios, with 95% confidence intervals in brackets. Standard errors are clustered at the household level

Significance levels: < 0.05*, < 0.01**, < 0.001***

*Abbreviations*: *ref.* reference category, *TCALE*  total cumulative adverse life event score, *SOCALE*  self-oriented cumulative adverse life event score, *OOCALE*  other-oriented cumulative adverse life event score, *UK*  United Kingdom

**Fig. 1:**
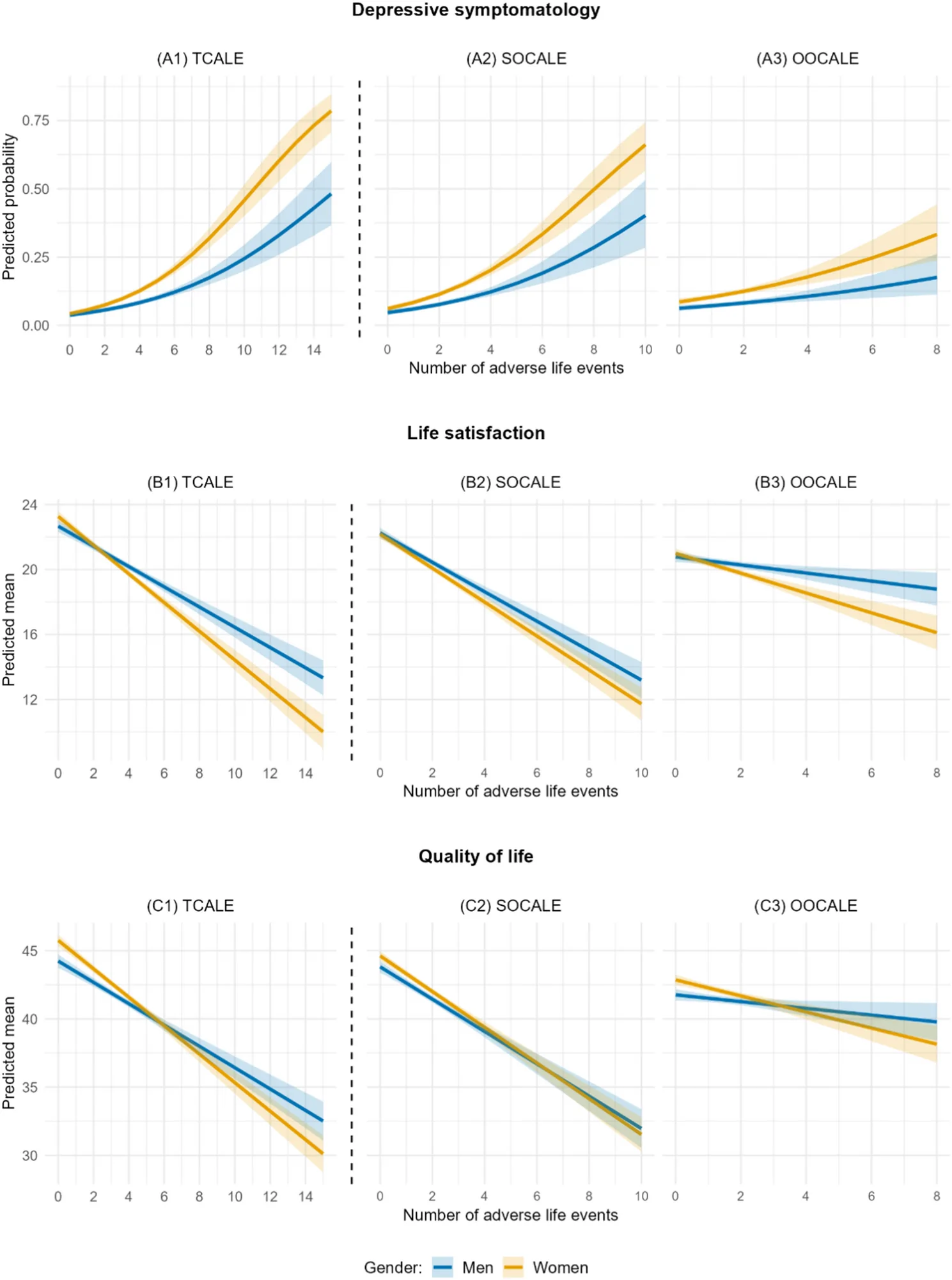
Predicted outcomes for depressive symptomatology, life satisfaction, and quality of life by gender. Notes: Figures display predicted probabilities for depressive symptomatology and predicted means for life satisfaction and quality of life based on Model 2 for TCALE and Model 4 for OOCALE and SOCALE, with 84% confidence intervals. Predicted outcomes represent average marginal predictions calculated over the observed distribution of all covariates. Standard errors are clustered at the household level. The dashed vertical line separates Model 2 (TCALE) from Model 4 (SOCALE, OOCALE). *Abbreviations:* *TCALE* Total cumulative adverse life event score, *SOCALE*  Self-oriented cumulative adverse life event score, *OOCALE*  Other-oriented cumulative adverse life event score

The association between TCALE and depressive symptomatology is significantly moderated by gender (OR = 1.09, 95% CI: [1.00; 1.18]; Model 3), with women showing a steeper increase in predicted probability of depressive symptomatology than men as adverse life events accumulate (Fig. 1; Panel A1). No significant gender moderation is observed for SOCALE (OR = 1.08, 95% CI: [0.96; 1.22]; Model 4) and OOCALE (OR = 1.07, 95% CI: [0.92; 1.24]; Model 4). Nevertheless, the predicted probabilities presented in Fig. 1 (Panels A2 and A3) show non-significant trends, indicating that the accumulation of both self- and other-oriented adverse life events may be somewhat more strongly associated with depressive symptomatology in women compared to men.

Table 5 displays the regression results for the relationship between the three accumulation scores, TCALE, SOCALE, and OOCALE, with life satisfaction. Each additional adverse life event is associated with 0.75 scale points lower life satisfaction (β = -0.75, 95% CI: [-0.85; -0.66]; Model 1). When considering event orientation, each additional self-oriented event corresponds to 0.98 scale points lower life satisfaction (β = -0.98, 95% CI: [-1.11; -0.85]; Model 2), whereas each additional other-oriented event is associated with 0.42 scale points lower life satisfaction (β = -0.42, 95% CI: [-0.58; -0.26]; Model 2). Significant moderation effects of gender are observed for TCALE (β = -0.26, 95% CI: [-0.43; -0.09]; Model 3) and OOCALE (β = -0.36, 95% CI: [-0.66; -0.06]; Model 4), indicating that the negative association between adverse life event accumulation and life satisfaction is stronger in women. As shown in Fig. 1 (Panels B1 and B3), women report slightly higher life satisfaction at lower levels of TCALE and OOCALE, yet show a steeper gradient across higher levels of accumulated adversity compared to men. No significant gender moderation is found for SOCALE (β = -0.14, 95% CI: [-0.38; 0.10]; Model 4), although Fig. 1 (Panel B2) shows a comparable non-significant trend to the ones of TCALE and OOCALE.

**Table 5:** Linear regression models predicting life satisfaction

	Model 1	Model 2	Model 3	Model 4
TCALE	-0.75 *** [-0.85; -0.66]	-	-0.62 *** [-0.75; -0.49]	-
SOCALE	-	-0.98 *** [-1.11; -0.85]	-	-0.91 *** [-1.10; -0.72]
OOCALE	-	-0.42 *** [-0.58; -0.26]	-	-0.25 * [-0.47; -0.03]
TCALE x Gender	-	-	-0.26 ** [-0.43; -0.09]	-
SOCALE x Gender	-	-	-	-0.14 [-0.38; 0.10]
OOCALE x Gender	-	-	-	-0.36 * [-0.66; -0.06]
Gender: Men (ref.)	-	-	-	-
Women	-0.37 * [-0.70; -0.05]	-0.37 * [-0.69; -0.05]	0.60 [-0.06; 1.25]	0.50 [-0.15; 1.16]
Age: ≤ 59 years (ref.)	-	-	-	-
60–69 years	0.57 * [0.13; 1.02]	0.72 ** [0.27; 1.17]	0.58 * [0.13; 1.02]	0.72 ** [0.27; 1.17]
70–79 years	1.58 *** [1.12; 2.05]	1.70 *** [1.24; 2.17]	1.57 *** [1.11; 2.03]	1.69 *** [1.23; 2.16]
80 + years	1.28 *** [0.62; 1.95]	1.21 *** [0.56; 1.87]	1.26 *** [0.60; 1.92]	1.20 *** [0.55; 1.86]
Birthplace: UK (ref.)	-	-	-	-
Outside of UK	0.22 [-0.53; 0.98]	0.13 [-0.62; 0.88]	0.21 [-0.55; 0.96]	0.12 [-0.63; 0.87]
Highest education: No qualification (ref.)	-	-	-	-
Low education	-0.17 [-0.63; 0.29]	-0.20 [-0.66; 0.26]	-0.18 [-0.64; 0.28]	-0.21 [-0.67; 0.25]
Medium education	0.38 [-0.13; 0.89]	0.31 [-0.20; 0.82]	0.38 [-0.13; 0.89]	0.30 [-0.21; 0.81]
High education	0.87 ** [0.32; 1.42]	0.81 ** [0.26; 1.36]	0.89 ** [0.34; 1.43]	0.82 ** [0.27; 1.36]
Intercept	22.29 *** [21.68; 22.90]	22.18 *** [21.57; 22.79]	21.78 *** [21.10; 22.46]	21.75 *** [21.07; 22.42]

Shown values are beta coefficients, with 95% confidence intervals in brackets. Standard errors are clustered at the household level

Significance levels: < 0.05*, < 0.01**, < 0.001***

*Abbreviations*: *ref.* reference category, *TCALE*  total cumulative adverse life event score, *SOCALE*  self-oriented cumulative adverse life event score, *OOCALE*  other-oriented cumulative adverse life event score, *UK*  United Kingdom

Finally, Table 6 presents regression results for the association between the accumulation scores TCALE, SOCALE, and OOCALE, and quality of life. Each additional adverse life event is associated with 0.91 scale points lower quality of life (β = -0.91, 95% CI: [-1.03; -0.80]; Model 1). Distinguishing by event orientation of adverse life events, each additional self-oriented adverse life event is associated with 1.25 scale points lower quality of life (β = -1.25, 95% CI: [-1.41; -1.09]; Model 2), whereas each additional other-oriented adverse life event is associated with 0.41 scale points lower quality of life (β = -0.41, 95% CI: [-0.62; -0.20]; Model 2).

A significant interaction term between TCALE and gender is observed (β = -0.26, 95% CI: [-0.49; -0.04]; Model 3), indicating that the negative association between cumulative adverse life events and quality of life is stronger in women. No significant gender differences are found for SOCALE (β = -0.12, 95% CI: [-0.44; 0.19]; Model 4) and OOCALE (β = -0.34, 95% CI: [-0.74; 0.06]; Model 4), although the interaction term for OOCALE only narrowly misses significance. As illustrated in Fig. 1 (Panel C3), women report higher quality of life at lower levels of OOCALE yet show a steeper gradient across higher levels of accumulated adversity compared to men.

**Table 6:** Linear regression models predicting quality of life

	Model 1	Model 2	Model 3	Model 4
TCALE	-0.91 *** [-1.03; -0.80]	-	-0.78 *** [-0.95; -0.61]	-
SOCALE	-	-1.25 *** [-1.41; -1.09]	-	-1.18 *** [-1.43; -0.94]
OOCALE	-	-0.41 *** [-0.62; -0.20]	-	-0.25 [-0.54; 0.05]
TCALE x Gender	-	-	-0.26 * [-0.49; -0.04]	-
SOCALE x Gender	-	-	-	-0.12 [-0.44; 0.19]
OOCALE x Gender	-	-	-	-0.34 [-0.74; 0.06]
Gender: Men (ref.)	-	-	-	-
Women	0.54 * [0.12; 0.97]	0.54 * [0.12; 0.97]	1.51 *** [0.64; 2.39]	1.36 ** [0.48; 2.24]
Age: ≤ 59 years (ref.)	-	-	-	-
60–69 years	0.55 [-0.02; 1.12]	0.77 ** [0.20; 1.34]	0.55 [-0.02; 1.12]	0.77 ** [0.19; 1.34]
70–79 years	0.21 [-0.41; 0.83]	0.39 [-0.23; 1.01]	0.19 [-0.43; 0.81]	0.38 [-0.24; 1.00]
80 + years	-2.14 *** [-3.03; -1.25]	-2.25 *** [-3.14; -1.37]	-2.17 *** [-3.06; -1.28]	-2.26 *** [-3.15; -1.38]
Birthplace: UK (ref.)	-	-	-	-
Outside of UK	-0.61 [-1.58; 0.36]	-0.75 [-1.72; 0.22]	-0.63 [-1.60; 0.34]	-0.76 [-1.73; 0.21]
Highest education: No qualification (ref.)	-	-	-	-
Low education	1.43 *** [0.80; 2.06]	1.39 *** [0.77; 2.02]	1.43 *** [0.80; 2.05]	1.38 *** [0.76; 2.01]
Medium education	2.70 *** [2.02; 3.39]	2.59 *** [1.91; 3.27]	2.70 *** [2.02; 3.39]	2.58 *** [1.90; 3.27]
High education	3.86 *** [3.15; 4.57]	3.77 *** [3.06; 4.48]	3.88 *** [3.16; 4.59]	3.78 *** [3.06; 4.49]
Intercept	42.85 *** [42.04; 43.66]	42.69 *** [41.88; 43.50]	42.34 *** [41.43; 43.26]	42.29 *** [41.38; 43.20]

Shown values are beta coefficients, with 95% confidence intervals in brackets. Standard errors are clustered at the household level

Significance levels: < 0.05*, < 0.01**, < 0.001***

*Abbreviations*: *ref.* reference category, *TCALE*  total cumulative adverse life event score, *SOCALE*  self-oriented cumulative adverse life event score, *OOCALE*  other-oriented cumulative adverse life event score, *UK*  United Kingdom

## Discussion

### Summary of findings

This study examined whether the accumulation of adverse life events across the life course – overall and differentiated by event orientation – is associated with depressive symptomatology, life satisfaction, and quality of life, as well as potential gender differences in these relationships.

Results show that greater accumulation of adverse life events, overall and separately for self- and other-oriented adverse events, is linked to a higher risk of depressive symptomatology, lower life satisfaction, and poorer quality of life. Gender moderates several of these associations. Women appear to be more vulnerable to the overall accumulation of adverse life events with respect to all three outcomes, as well as to the accumulation of other-oriented adverse life events in relation to life satisfaction. For depressive symptomatology and quality of life, women likewise tend to show poorer outcomes as other-oriented events accumulate compared with men, although these gender differences do not reach statistical significance. This study found no clear evidence for gender differences in the associations between accumulated self-oriented adverse life events and any of the outcomes examined.

The observed relationship between the accumulation of adverse life events and poorer mental health and reduced well-being is consistent with prior research [1, 2, 18, 48–59]. This study contributes to the existing literature by confirming these associations while considering a wide range of adverse events. Results for self-oriented adverse life events also align with existing evidence showing that the accumulation of direct personal adversity is associated with poorer mental health and well-being [18, 55, 56, 60]. Importantly, the present findings also indicate that the accumulation of other-oriented adverse life events is related to a higher risk of depressive symptomatology, lower life satisfaction, and reduced quality of life, contrasting with previous studies reporting no association [55, 56, 60] or even beneficial effects of accumulated other-oriented adversity for mental health and well-being [56, 60].

One possible explanation for these divergent findings on the consequences of accumulated other-oriented adverse life events is contextual differences between study populations. Previous studies reporting contrasting findings were conducted among older Israeli adults [55, 56, 60]. This population is characterized by exceptionally high lifetime exposure to collective stressors and trauma, including the Holocaust, large-scale migration, and repeated military conflicts [61, 62]. Correspondingly, the prevalence of depression in this population is nearly twice as high as in Western countries [55], such as England. Such extensive exposure to severe and collective stressors may shape both the experience and psychological processing of adverse life events in ways that differ substantially from those in other national contexts [55]. Compared with previous work examining consequences of accumulated other-oriented adverse life events for mental health and well-being in England [18], the present study covers a broader spectrum of other-oriented adverse events and provides additional evidence that their accumulation is associated with poorer mental health and reduced well-being.

The central contribution of this study is the examination of gender differences in the relationship between the accumulation of adverse life events and mental health and well-being. Results indicate a greater vulnerability to overall adverse life event accumulation with respect to all outcomes, and thus across a wide range of well-being dimensions, among women compared to men. This finding is consistent with prior evidence suggesting heightened sensitivity in women to accumulated adverse life events in relation to depression [52]. The present findings extend this existing evidence by showing that these gendered patterns also apply to other facets of well-being, namely life satisfaction and quality of life. Importantly, by distinguishing between self- and other-oriented adverse life events, this study provides novel insight into how observed gendered patterns vary depending on the orientation of accumulated adverse events.

Regarding the accumulation of other-oriented adverse life events, gender differences were visible, reaching statistical significance for life satisfaction while narrowly missing significance for depressive symptomatology and quality of life. This indicates that gendered consequences of the accumulation of other-oriented adverse life events are particularly pronounced for subjective evaluations of one’s life circumstances. Given that associations clearly and consistently point in the same direction across outcomes, the non-significant results for accumulated other-oriented adverse life events may reflect insufficient statistical power to detect small but potentially meaningful gender differences rather than a true absence of variation by gender.

The lack of significant gender differences in the relationship between accumulated self-oriented adverse events and mental health and well-being may reflect the nature of these stressors. Self-oriented adverse events are primarily processed through personal coping resources [140] and are often perceived as less controllable than other-oriented adverse events [72, 141]. This could reduce the influence of gender-role expectations in the coping process. The relative uncontrollability of self-oriented events also enhances the effectiveness of emotion-focused coping strategies [142], which women tend to employ more than men [82, 90–93], potentially buffering the negative consequences of accumulated self-oriented events for women’s mental health and well-being and thereby attenuating observable gender differences for this event type.

### Strengths, limitations, and further research directions

A major strength of this study is the broad range of adverse life events considered. However, information on some potentially important events, such as the death of a sibling, was not available, and other events, such as unexpected job loss, are only partially captured with ELSA data. Some of these unobserved adverse events may differ in their consequences for men’s and women’s mental health and well-being [72, 141]. As a result, the estimated associations and observed gender differences reflect the set of adverse life events covered in this study.

Due to limited information on event frequency for most adverse life events, accumulation was operationalized as the diversity of events experienced over the life course. While this checklist-based approach does not capture repeated exposure to adverse events, it nevertheless provides a widely used and transparent measure of cumulative adversity [1]. Furthermore, although relying on a unweighted sum score is the most commonly used approach for measuring the accumulation of adverse life events, it does not account for differences in the perceived severity of individual events nor for variations in their overall impact [132]. A strength of this study is that it addresses these two measurement-related concerns through two sensitivity analyses. First, frequency information for a subset of events was incorporated, and second, event weights derived from an external study [133] were applied. Both additional analyses yielded results largely consistent with the main analysis, supporting the robustness of the findings. It should be noted, however, that the available weights did not perfectly correspond to every adverse life event considered in this study, were not specifically developed for the outcomes examined, and do not account for the timing of events in the life course, therefore providing only an approximate indication of the relative burden for each event.

As with all retrospective collection of information, recall bias cannot be entirely ruled out. Despite the use of a life history calendar in ELSA, improving recall accuracy [102, 143], some memory limitations remain, particularly for events that occurred decades before the interview [140, 144]. Gender differences in recall and reporting of adversities and mental health outcomes may also affect the results of this study. Women tend to remember and ruminate more about past adverse events than men [145–147], while men tend to report fewer events due to social norms encouraging emotional restraint, self-control, and stoicism [69, 71, 148]. In addition, internalized gender norms and concerns about negative social perceptions may affect men’s willingness to disclose mental health difficulties [149], although some research has found no gender differences in the reporting of depressive symptoms [150, 151]. While these patterns could influence observed gender differences, the direction and magnitude of any resulting bias remain uncertain.

Gender was operationalized as a binary variable, consistent with ELSA coding. Yet gender is a multidimensional construct encompassing non-binary, trans, and gender-diverse identities [152]. Future research incorporating more inclusive measures of gender may provide more nuanced patterns.

Although temporal ordering is supported by the timing of events preceding outcome assessment, causal interpretations remain limited due to the cross-sectional study design [153]. In addition, information on participants’ characteristics prior to or at the time of each event, such as social engagement, socioeconomic status, and childhood socioeconomic circumstances, was not available, limiting adjustment for potential confounders [63, 154]. Yet, results proved to be largely robust to the inclusion of available additional confounding variables, supporting the robustness of the findings.

The data used in this study was collected in 2006/2007 and therefore reflects the social, healthcare, and gender norms of that period. Societal changes since then, including reduced stigma toward mental illness [155], shifts toward more egalitarian gender roles [156, 157], and improvements in social support and healthcare structures [158, 159], should be considered when interpreting the findings in a contemporary context. In addition, most participants experienced the Second World War and/or the post-war period, followed by considerable economic and social changes in the UK, which may have shaped coping strategies and responses to adversity [160]. While participants born after 1945 account for approximately 50% of the sample, most were born in the late 1940s and early 1950s and thus still grew up in the immediate post-war period. As those born sufficiently late to be largely unaffected by wartime and its direct aftermath represent only a very small fraction of the study sample, subgroup analyses for this group were not feasible.

In addition, findings of this study reflect the experiences of adults aged 50 and older and their partners living in private households in England, as represented in the ELSA study [100]. Their life circumstances have been shown to differ from those of younger age groups [93] and people living outside traditional private households [161]. It has been shown that coping behaviors change across the life course, with evidence indicating a convergence between men and women in the use of coping strategies as they age, with both increasingly relying on problem-focused coping [93]. Therefore, further research is needed to determine the extent to which these findings generalize to other cohorts, younger age groups, and different living arrangements.

The analysis sample included about 52% of the original Wave 3 ELSA participants, as many did not complete the life history interview or self-completion questionnaires or had missing information in important study variables. Differences between included and excluded participants (presented in Additional File 1) suggest potential selection effects, which should be taken into account when interpreting the findings of this research.

Future research should further examine the consequences of accumulating adverse life events for mental health and well-being, and especially associated gender differences, by extending analyses to populations of different ages, cultural contexts, and living arrangements (e.g., including individuals living in institutional settings such as nursing homes). Applying alternative approaches to capture the accumulation of adverse life events, such as taking into account repeated exposure, perceived burden, and different sets of adverse life events, may further advance the insights gained from this study.

## Conclusion

The results of this study indicate that the accumulation of adverse life events across the life course, overall and separately for self- and other-oriented events, is associated with a higher risk of experiencing depressive symptomatology, lower life satisfaction, and reduced quality of life. The consistent associations across all three outcomes suggest that cumulative adversity represents a substantial source of strain across multiple dimensions of psychological well-being. Women’s mental health and well-being appear more vulnerable to cumulative adversity compared to men’s, particularly regarding overall accumulation and the accumulation of other-oriented adverse life events.

From a public health perspective, these findings underscore the importance of identifying populations with high cumulative adversity exposure, with particular attention to women who may be particularly affected by its long-term consequences. Gender-sensitive interventions, including targeted mental health support, promotion of coping resources, and strategies addressing both the overall burden and orientation of adverse events, may help mitigate long-term negative consequences of accumulated adversity for mental health and well-being.

Future research should build on these findings by examining gendered consequences of cumulative adversity for mental health and well-being with greater attention to frequency and perceived severity, as well as across different populations.

## Supplementary Information

Additional file 1: Comparison of included vs. excluded participants. This file contains results of comparisons between individuals included in the analytical sample and those excluded due to non-participation in the life history interview, non-completion of the self-completion questionnaire, or missing data, in order to evaluate potential selection bias. The comparison is based on key characteristics considered in both the main and sensitivity analyses.

Additional file 2: Testing for non-linear effects of cumulative adverse life events on mental health and well-being. This file contains the results of regression models, including quadratic terms and smoothing splines for the three accumulation scores across all outcomes, used to assess potential non-linear associations.

Additional file 3: Gender-stratified regression models. This file contains the results of the main analysis regression models estimated separately for men and women.

Additional file 4: Weights assigned to individual adverse life events for sensitivity analysis. This file contains the weights assigned to each adverse life event based on perceived stress and adjustment demands, used in the second sensitivity analysis to calculate a weighted cumulative adversity score.

Additional file 5: Results of sensitivity analyses. This file contains descriptive statistics for variables where values or patterns differed from the main analysis, as well as the regression results for all four sensitivity analyses.

Additional file 6: Additional descriptive information on accumulation scores and well-being outcomes. This file contains additional descriptive statistics comprising the 25th percentile, median, and 75th percentile of the three accumulation scores, the life satisfaction score, and the quality of life score.

## Data Availability

English Longitudinal Study of Ageing (ELSA) data can be accessed by application via https://www.elsa-project.ac.uk/accessing-elsa-data.

## Abbreviations

CASP-19: Control, Autonomy, Self-realization, and Pleasure 19
CASP-12: Control, Autonomy, Self-realization, and Pleasure 12
CES-D: Center for Epidemiological Studies Depression Scale
CLAM: Cumulative Lifetime Adversity Measure
ELSA: English Longitudinal Study of Ageing
OOCALE: Other-oriented cumulative adverse life event score
SOCALE: Self-oriented cumulative adverse life event score
TCALE: Total cumulative adverse life event score

## References

[1] Bossert SA, Jayawickreme E, Blackie LER, Cole VT. Further exploring the impact of cumulative lifetime adversity on life satisfaction, psychological flourishing, and depressive symptoms. J Res Personal. 2024;110:104488. 10.1016/j.jrp.2024.104488.

[2] Shrira A. The effect of lifetime cumulative adversity on change and chronicity in depressive symptoms and quality of life in older adults. Int Psychogeriatr. 2012;24(12):1988–97. 10.1017/S1041610212001123. 22874666

[3] Berlemann M, Eurich M. Natural disasters and self-reported well-being: a review of the literature. In: Handbook on the Economics of Disasters [Internet]. Edward Elgar Publishing; 2022 [cited 2025 Dec 17]. pp. 230–51. (Chapters). Available from: https://EconPapers.repec.org/RePEc:elg:eechap:19599_12

[4] Carey KB, Norris AL, Durney SE, Shepardson RL, Carey MP. Mental health consequences of sexual assault among first-year college women. J Am Coll Health. 2018;66(6):480–6. 10.1080/07448481.2018.1431915. PMC6311089 29405862

[5] Keyes KM, Pratt C, Galea S, McLaughlin KA, Koenen KC, Shear MK. The Burden of Loss: Unexpected Death of a Loved One and Psychiatric Disorders Across the Life Course in a National Study. Am J Psychiatry. 2014;171(8):864–71. 10.1176/appi.ajp.2014.13081132. PMC4119479 24832609

[6] Liu WM, Forbat L, Anderson K, Correction. Death of a close friend: Short and long-term impacts on physical, psychological and social well-being. PLoS ONE. 2019;14(5):e0218026. 10.1371/journal.pone.0218026. PMC6448887 30947290

[7] McGuire AP, Gauthier JM, Anderson LM, Hollingsworth DW, Tracy M, Galea S, et al. Social Support Moderates Effects of Natural Disaster Exposure on Depression and Posttraumatic Stress Disorder Symptoms: Effects for Displaced and Nondisplaced Residents. J Trauma Stress. 2018;31(2):223–33. 10.1002/jts.22270. PMC6020825 29623684

[8] Morton KR, Tanzini L, Lee JW. Adult Life Satisfaction and the Role of Forgiveness After Childhood Sexual Abuse: Evidence from a Seventh-day Adventist Cohort. J Sci Study Relig. 2019;58(1):138–52. 10.1111/jssr.12575.

[9] Sbarra DA. Divorce and Health: Current Trends and Future Directions. Psychosom Med. 2015;77(3):227–36. 10.1097/PSY.0000000000000168. PMC4397145 25829240

[10] Van Scheppingen MA, Leopold T. Trajectories of life satisfaction before, upon, and after divorce: Evidence from a new matching approach. J Pers Soc Psychol. 2020;119(6):1444–58. 10.1037/pspp0000270. 31580108

[11] Thomas S. Dimensions of family disruption: Coincidence, interactions, and impacts on children’s educational attainment. Longitud Life Course Stud. 2018;9(2):157–87. 10.14301/llcs.v9i2.436.

[12] Albuquerque S, Pereira M, Narciso I. Couple’s Relationship After the Death of a Child: A Systematic Review. J Child Fam Stud. 2016;25(1):30–53. 10.1007/s10826-015-0219-2.

[13] Clark WAV. Life events and moves under duress: disruption in the life course and mobility outcomes. Longitud Life Course Stud. 2016;7(3). 10.14301/llcs.v7i3.376.

[14] Di Nallo A, Lipps O, Oesch D, Voorpostel M. The effect of unemployment on couples separating in Germany and the UK. J Marriage Fam. 2022;84(1):310–29. 10.1111/jomf.12803.

[15] Comolli CL, Bolano D, Bernardi L, Voorpostel M. Concentration of critical events over the life course and life satisfaction later in life. Adv Life Course Res. 2024;61:100616. 10.1016/j.alcr.2024.100616. 38759484

[16] Pearlin LI, Schieman S, Fazio EM, Meersman SC. Stress, Health, and the Life Course: Some Conceptual Perspectives. J Health Soc Behav. 2005;46(2):205–19. 10.1177/002214650504600206. 16028458

[17] Bøe T, Serlachius AS, Sivertsen B, Petrie KJ, Hysing M. Cumulative effects of negative life events and family stress on children’s mental health: the Bergen Child Study. Soc Psychiatry Psychiatr Epidemiol. 2018;53(1):1–9. 10.1007/s00127-017-1451-4. 29090324

[18] Richardson S, Carr E, Netuveli G, Sacker A. Adverse events over the life course and later-life wellbeing and depressive symptoms in older people. Int Psychogeriatr. 2023;35(5):243–57. 10.1017/S1041610220003373. 33050971

[19] Shrira A, Litwin H. The Effect of Lifetime Cumulative Adversity and Depressive Symptoms on Functional Status. J Gerontol B Psychol Sci Soc Sci. 2014;69(6):953–65. 10.1093/geronb/gbu056. PMC4296139 24898028

[20] Kessler RC. The Effects of Stressful Life Events on Depression. Annu Rev Psychol. 1997;48(1):191–214. 10.1146/annurev.psych.48.1.191. 9046559

[21] Fiske A, Wetherell JL, Gatz M. Depression in Older Adults. Annu Rev Clin Psychol. 2009;5(1):363–89. 10.1146/annurev.clinpsy.032408.153621. PMC2852580 19327033

[22] Patel V, Chisholm D, Parikh R, Charlson FJ, Degenhardt L, Dua T, et al. Addressing the burden of mental, neurological, and substance use disorders: key messages from Disease Control Priorities, 3rd edition. Lancet. 2016;387(10028):1672–85. 10.1016/S0140-6736(15)00390-6. 26454360

[23] Goodwin RD, Dierker LC, Wu M, Galea S, Hoven CW, Weinberger AH. Trends in U.S. Depression Prevalence From 2015 to 2020: The Widening Treatment Gap. Am J Prev Med. 2022;63(5):726–33. 10.1016/j.amepre.2022.05.014. PMC9483000 36272761

[24] Santomauro DF, Mantilla Herrera AM, Shadid J, Zheng P, Ashbaugh C, Pigott DM, et al. Global prevalence and burden of depressive and anxiety disorders in 204 countries and territories in 2020 due to the COVID-19 pandemic. Lancet. 2021;398(10312):1700–12. 10.1016/S0140-6736(21)02143-7. PMC8500697 34634250

[25] König H, König HH, Konnopka A. The excess costs of depression: a systematic review and meta-analysis. Epidemiol Psychiatr Sci. 2020;29:e30. 10.1017/S2045796019000180. PMC8061284 30947759

[26] Felce D, Perry J. Quality of life: Its definition and measurement. Res Dev Disabil. 1995;16(1):51–74. 10.1016/0891-4222(94)00028-8. 7701092

[27] Fergusson DM, McLeod GFH, Horwood LJ, Swain NR, Chapple S, Poulton R. Life satisfaction and mental health problems (18 to 35 years). Psychol Med. 2015;45(11):2427–36. 10.1017/S0033291715000422. 25804325

[28] Kim ES, Delaney SW, Tay L, Chen Y, Diener E, Vanderweele TJ. Life Satisfaction and Subsequent Physical, Behavioral, and Psychosocial Health in Older Adults. Milbank Q. 2021;99(1):209–39. 10.1111/1468-0009.12497. PMC7984669 33528047

[29] Strine TW, Chapman DP, Balluz LS, Moriarty DG, Mokdad AH. The Associations Between Life Satisfaction and Health-related Quality of Life, Chronic Illness, and Health Behaviors among U.S. Community-dwelling Adults. J Community Health. 2008;33(1):40–50. 10.1007/s10900-007-9066-4. 18080207

[30] Bayrak M, Çadirci K. The associations of life quality, depression, and cognitive impairment with mortality in older adults with COVID-19: a prospective, observational study. Acta Clin Belg. 2022;77(3):588–95. 10.1080/17843286.2021.1916687. 33870876

[31] Hohls JK, König HH, Quirke E, Hajek A, Anxiety. Depression and Quality of Life—A Systematic Review of Evidence from Longitudinal Observational Studies. Int J Environ Res Public Health. 2021;18(22):12022. 10.3390/ijerph182212022. PMC8621394 34831779

[32] Michalos AC, Zumbo BD, Hubley A. Health and the Quality of Life. Soc Indic Res. 2000;51(3):245–86. 10.1023/A:1007010401301.

[33] Boehm JK, Chen Y, Williams DR, Ryff CD, Kubzansky LD. Subjective well-being and cardiometabolic health: An 8–11year study of midlife adults. J Psychosom Res. 2016;85:1–8. 10.1016/j.jpsychores.2016.03.018. PMC4889157 27212662

[34] Martín-María N, Miret M, Caballero FF, Rico-Uribe LA, Steptoe A, Chatterji S, et al. The Impact of Subjective Well-being on Mortality: A Meta-Analysis of Longitudinal Studies in the General Population. Psychosom Med. 2017;79(5):565–75. 10.1097/PSY.0000000000000444. 28033196

[35] Phyo AZZ, Freak-Poli R, Craig H, Gasevic D, Stocks NP, Gonzalez-Chica DA, et al. Quality of life and mortality in the general population: a systematic review and meta-analysis. BMC Public Health. 2020;20(1):1596. 10.1186/s12889-020-09639-9. PMC7646076 33153441

[36] Simonova MD, Beyond GDP. Measuring What Counts for Economic and Social Performance. MGIMO Rev Int Relat. 2019;12(5):172–80. 10.24833/2071-8160-2019-5-68-172-180.

[37] Durand M, Exton C. Adopting a Well-Being Approach in Central Government: Policy Mechanisms and Practical Tools. In: Global Happiness and Well-Being Policy Report [Internet]. New York, NY: Sustainable Development Solutions Network; 2019 [cited 2025 Dec 17]. pp. 141–62. Available from: https://www.happinesscouncil.org/report/2019/global-happiness-and-well-being-policy-report

[38] Bernard JER, Depression. A Review of its Definition. MOJ Addict Med Ther. 2018;5(1):62–5. 10.15406/mojamt.2018.05.00082.

[39] Diener E, Emmons RA, Larsen RJ, Griffin S. The Satisfaction With Life Scale. J Pers Assess. 1985;49(1):71–5. 10.1207/s15327752jpa4901_13. 16367493

[40] Wheaton B. Sampling the Stress Universe. In: Avison WR, Gotlib IH, editors. Stress and Mental Health: Contemporary Issues and Prospects for the Future [Internet]. Boston, MA: Springer US; 1994 [cited 2025 Dec 17]. pp. 77–114. Available from: 10.1007/978-1-4899-1106-3_4 doi:10.1007/978-1-4899-1106-3_4.

[41] Holmes TH, Rahe RH. The social readjustment rating scale. J Psychosom Res. 1967;11(2):213–8. 10.1016/0022-3999(67)90010-4. 6059863

[42] American Psychiatric Association. Diagnostic and statistical manual of mental disorders, 4th ed. Arlington, VA, US: American Psychiatric Publishing, Inc. 1994. xxvii, 886 p. (Diagnostic and statistical manual of mental disorders, 4th ed).

[43] Filipp SH, Aymanns P. Kritische Lebensereignisse und Lebenskrisen [Internet]. W. Kohlhammer GmbH; 2018 [cited 2025 Dec 17]. Available from: https://elibrary.kohlhammer.de/book/10.17433/

[44] Hammen C, Kim EY, Eberhart NK, Brennan PA. Chronic and Acute Stress and the Prediction of Major Depression in Women. Depress Anxiety. 2009;26(8):718–23. 10.1002/da.20571 . PubMed PMID: 19496077; PubMed Central PMCID: PMC3380803. PMC3380803 19496077

[45] Elder GH. The Life Course as Developmental Theory. Child Dev. 1998;69(1):1. 10.2307/1132065. 9499552

[46] Bengtson VL, Elder GH, Putney NM. The Lifecourse Perspective on Ageing: Linked Lives, Timing, and History. In: Johnson ML, Bengtson VL, Coleman PG, Kirkwood TBL, editors. The Cambridge Handbook of Age and Ageing [Internet]. 1st ed. Cambridge University Press; 2005 [cited 2025 Dec 17]. pp. 493–501. Available from: https://www.cambridge.org/core/product/identifier/9780511610714%23c82632-3230a/type/book_part

[47] Ferraro KF, Shippee TP. Aging and Cumulative Inequality: How Does Inequality Get Under the Skin? Gerontologist. 2009;49(3):333–43. 10.1093/geront/gnp034. PMC2721665 19377044

[48] Abravanel BT, Sinha R. Emotion dysregulation mediates the relationship between lifetime cumulative adversity and depressive symptomatology. J Psychiatr Res. 2015;61:89–96. 10.1016/j.jpsychires.2014.11.012. PMC4308421 25528603

[49] Ahn S, Kim S, Zhang H, Dobalian A, Slavich GM. Lifetime adversity predicts depression, anxiety, and cognitive impairment in a nationally representative sample of older adults in the United States. J Clin Psychol. 2024;80(5):1031–49. 10.1002/jclp.23642. PMC11216061 38294127

[50] Dalgard OS, Dowrick C, Lehtinen V, Vazquez-Barquero JL, Casey P, Wilkinson G, et al. Negative life events, social support and gender difference in depression: A multinational community survey with data from the ODIN study. Soc Psychiatry Psychiatr Epidemiol. 2006;41(6):444–51. 10.1007/s00127-006-0051-5. 16572275

[51] Dulin PL, Passmore T. Avoidance of potentially traumatic stimuli mediates the relationship between accumulated lifetime trauma and late-life depression and anxiety. J Trauma Stress. 2010;23(2):296–9. 10.1002/jts.20512. 20419741

[52] Falkingham J, Evandrou M, Qin M, Vlachantoni A. Accumulated lifecourse adversities and depressive symptoms in later life among older men and women in England: a longitudinal study. Ageing Soc. 2020;40(10):2079–105. 10.1017/S0144686X19000461.

[53] Infurna FJ, Staben OE, Gardner MJ, Grimm KJ, Luthar SS. The accumulation of adversity in midlife: Effects on depressive symptoms, life satisfaction, and character strengths. Psychol Aging. 2023;38(3):230–46. 10.1037/pag0000725. 36795424

[54] Kok AAL, Twisk JWR, Blom F, Beekman ATF, Huisman M. Steeling or Sensitizing? A Longitudinal Examination of How Ongoing Accumulation of Negative Life Events Affects Depressive Symptoms in Older Adults. Neupert S, editor. J Gerontol Ser B. 2021;76(10):2041–53. 10.1093/geronb/gbab114 PMC8599083 34171092

[55] Shmotkin D, Litwin H. Cumulative adversity and depressive symptoms among older adults in Israel: the differential roles of self-oriented versus other-oriented events of potential trauma. Soc Psychiatry Psychiatr Epidemiol. 2009;44(11):989–97. 10.1007/s00127-009-0020-x. PMC3547167 19288036

[56] Shrira A, Shmotkin D, Litwin H. Potentially traumatic events at different points in the life span and mental health: Findings from SHARE-Israel. Am J Orthopsychiatry. 2012;82(2):251–9. 10.1111/j.1939-0025.2012.01149.x. PMC3538172 22506527

[57] Tibubos AN, Burghardt J, Klein EM, Brähler E, Jünger C, Michal M, et al. Frequency of stressful life events and associations with mental health and general subjective health in the general population. J Public Health. 2021;29(5):1071–80. 10.1007/s10389-020-01204-3.

[58] Turner RJ, Lloyd DA. Lifetime traumas and mental health: the significance of cumulative adversity. J Health Soc Behav. 1995;36(4):360–76. PubMed PMID: 8719054. 8719054

[59] Seery MD, Holman EA, Silver RC. Whatever does not kill us: Cumulative lifetime adversity, vulnerability, and resilience. J Pers Soc Psychol. 2010;99(6):1025–41. 10.1037/a0021344. 20939649

[60] Keinan G, Shrira A, Shmotkin D. The association between cumulative adversity and mental health: considering dose and primary focus of adversity. Qual Life Res. 2012;21(7):1149–58. 10.1007/s11136-011-0035-0. 21983715

[61] Lomranz J. Long-term adaptation to traumatic stress in light of adult development and aging perspectives. Stress and coping in later-life families. Washington, DC, US: Hemisphere Publishing Corp; 1990. pp. 99–121. (Series in applied psychology).

[62] Shmotkin D, Shrira A, Goldberg S, Palgi Y. Resilience and Vulnerability Among Aging Holocaust Survivors and Their Families: An Intergenerational Overview. J Intergenerational Relatsh. 2011;9(1):7–21. 10.1080/15350770.2011.544202.

[63] Ogle CM, Rubin DC, Siegler IC. Cumulative exposure to traumatic events in older adults. Aging Ment Health. 2014;18(3):316–25. 10.1080/13607863.2013.832730. PMC3944195 24011223

[64] Busch IM, Moretti F, Purgato M, Barbui C, Wu AW, Rimondini M. Dealing With Adverse Events: A Meta-analysis on Second Victims’ Coping Strategies. J Patient Saf. 2020;16(2):e51–60. 10.1097/PTS.0000000000000661. 32168267

[65] Hökkä M, Aho AL. Negative Life Changes in the Lives of a Working-Age Widows and Widowers and their Families After the Death of a Spouse- a Qualitative Study. OMEGA - J Death Dying. 2025;00302228251328198. 10.1177/00302228251328198.40111178

[66] Staub E, Vollhardt J. Altruism born of suffering: The roots of caring and helping after victimization and other trauma. Am J Orthopsychiatry. 2008;78(3):267–80. 10.1037/a0014223. 19123746

[67] Rim S, Hansen J, Trope Y. What happens why? Psychological distance and focusing on causes versus consequences of events. J Pers Soc Psychol. 2013;104(3):457–72. 10.1037/a0031024. 23276264

[68] Becchetti L, Conzo G. The Gender Life Satisfaction/Depression Paradox. Soc Indic Res. 2022;160(1):35–113. 10.1007/s11205-021-02740-5. PMC8449750 34566237

[69] Seematter-Bagnoud L, Karmaniola A, Santos-Eggimann B. Adverse life events among community-dwelling persons aged 65–70 years: gender differences in occurrence and perceived psychological consequences. Soc Psychiatry Psychiatr Epidemiol. 2010;45(1):9–16. 10.1007/s00127-009-0035-3. 19305935

[70] Krämer MD, Rohrer JM, Lucas RE, Richter D. Life events and life satisfaction: Estimating effects of multiple life events in combined models. Eur J Personal. 2025;39(1):3–23. 10.1177/08902070241231017.

[71] Matud MP. Gender differences in stress and coping styles. Personal Individ Differ. 2004;37(7):1401–15. 10.1016/j.paid.2004.01.010.

[72] Wallace D, Cooper NR, Sel A, Russo R. The social readjustment rating scale: Updated and modernised. Olorunlana A, editor. PLOS ONE. 2023;18(12):e0295943. 10.1371/journal.pone.0295943 PMC10727443 38109368

[73] Yang K, Girgus JS. Are Women More Likely than Men Are to Care Excessively about Maintaining Positive Social Relationships? A Meta-Analytic Review of the Gender Difference in Sociotropy. Sex Roles. 2019;81(3–4):157–72. 10.1007/s11199-018-0980-y.

[74] Peterson M. What men and women value at work: Implications for workplace health. Gend Med. 2004;1(2):106–24. 10.1016/S1550-8579(04)80016-0. 16115589

[75] Cross SE, Madson L. Models of the self: Self-construals and gender. Psychol Bull. 1997;122(1):5–37. 10.1037/0033-2909.122.1.5. 9204777

[76] Martin CL, Ruble DN. A developmental perspective of self-construals and sex differences: Comment on Cross and Madson (1997). Psychol Bull. 1997;122(1):45–50. 10.1037/0033-2909.122.1.45. 9204779

[77] Bagley SL, Weaver TL, Buchanan TW. Sex differences in physiological and affective responses to stress in remitted depression. Physiol Behav. 2011;104(2):180–6. 10.1016/j.physbeh.2011.03.004. 21396947

[78] Casale S. Gender Differences in Self-esteem and Self‐confidence. In: Carducci BJ, Nave CS, Fabio A, Saklofske DH, Stough C, editors. The Wiley Encyclopedia of Personality and Individual Differences [Internet]. 1st ed. Wiley; 2020 [cited 2025 Dec 18]. pp. 185–9. Available from: https://onlinelibrary.wiley.com/doi/ doi:10.1002/9781119547174.ch208.

[79] Marsala MJ, Belfry S, Orange JB, Christie AD. Sex-Related Differences in Functional Fitness Outcomes in Older Adults. J Aging Phys Act. 2023;31(4):556–67. 10.1123/japa.2022-0071. 36626909

[80] Mayra M. The Effect of Gender & Educational Level on Optimism, Self-Efficacy and Coping Responses of College and University Students. J Sci Enq. 2022;2(1). 10.54280/jse.222118.

[81] Paganini A, Persson M, Mark H. Influence of Gender, Dispositional Optimism, and Coping Strategies on Appearance-Related Distress Among Swedish Adults With Cleft Lip and Palate. Cleft Palate Craniofac J. 2022;59(6):715–23. 10.1177/10556656211025196. PMC9121524 34137296

[82] Veisani Y, Jalilian Z, Sadeghifard YZ, Mohamadian F. Association between common stressful life events and coping strategies in adults. J Educ Health Promot. 2021;10(1). 10.4103/jehp.jehp_519_20. PMC8459853 34667807

[83] Merluzzi J, Dobrev SD. Unequal on top: Gender profiling and the income gap among high earner male and female professionals. Soc Sci Res. 2015;53:45–58. 10.1016/j.ssresearch.2015.04.010. 26188437

[84] Schneebaum A, Rehm M, Mader K, Hollan K. The Gender Wealth Gap Across European Countries. Rev Income Wealth. 2018;64(2):295–331. 10.1111/roiw.12281.

[85] Cohn-Schwartz E, Schmitz A. From attitudes to social networks: National gender-role attitudes and gender differences in late-life social relationships. Soc Netw. 2024;76:79–87. 10.1016/j.socnet.2023.08.001.

[86] Cornwell B, Laumann EO, Schumm LP. The Social Connectedness of Older Adults: A National Profile. Am Sociol Rev. 2008;73(2):185–203. 10.1177/000312240807300201. PMC2583428 19018292

[87] Davis MI, Jason LA. Sex Differences in Social Support and Self-Efficacy Within a Recovery Community. Am J Community Psychol. 2005;36(3–4):259–74. 10.1007/s10464-005-8625-z. 16389499

[88] Milner A, Krnjacki L, LaMontagne AD. Age and gender differences in the influence of social support on mental health: a longitudinal fixed-effects analysis using 13 annual waves of the HILDA cohort. Public Health. 2016;140:172–8. 10.1016/j.puhe.2016.06.029. 27527844

[89] Kendler KS, Myers J, Prescott CA. Sex Differences in the Relationship Between Social Support and Risk for Major Depression: A Longitudinal Study of Opposite-Sex Twin Pairs. Am J Psychiatry. 2005;162(2):250–6. 10.1176/appi.ajp.162.2.250. 15677587

[90] Ando’ A, Giromini L, Ales F, Zennaro A. A multimethod assessment to study the relationship between rumination and gender differences. Scand J Psychol. 2020;61(6):740–50. 10.1111/sjop.12666. 32686855

[91] Panayiotou G, Karekla M, Leonidou C. Coping through avoidance may explain gender disparities in anxiety. J Context Behav Sci. 2017;6(2):215–20. 10.1016/j.jcbs.2017.04.005.

[92] Baker JP, Berenbaum H. Emotional approach and problem-focused coping: A comparison of potentially adaptive strategies. Cogn Emot. 2007;21(1):95–118. 10.1080/02699930600562276.

[93] Nolen-Hoeksema S, Aldao A. Gender and age differences in emotion regulation strategies and their relationship to depressive symptoms. Personal Individ Differ. 2011;51(6):704–8. 10.1016/j.paid.2011.06.012.

[94] Garnefski N, Kraaij V, Spinhoven P. Negative life events, cognitive emotion regulation and emotional problems. Personal Individ Differ. 2001;30(8):1311–27. 10.1016/S0191-8869(00)00113-6.

[95] Kashdan TB, Barrios V, Forsyth JP, Steger MF. Experiential avoidance as a generalized psychological vulnerability: Comparisons with coping and emotion regulation strategies. Behav Res Ther. 2006;44(9):1301–20. 10.1016/j.brat.2005.10.003. 16321362

[96] Pekkarinen T. Gender Differences in Education [Internet]. IZA - Institute of Labor Economics; 2012 [cited 2026 Sep 18]. Available from: https://www.jstor.org/stable/resrep74563

[97] Cerise S. Accumulating poverty? Women’s experiences of inequality over the lifecycle. Sydney: Australian Human Rights Commission; 2009.

[98] Ice E. Bringing Family Demography Back In: A Life Course Approach to the Gender Gap in Caregiving in the United States. Soc Forces. 2023;101(3):1143–70. 10.1093/sf/soac041.

[99] Kiely KM, Brady B, Byles J. Gender, mental health and ageing. Maturitas. 2019;129:76–84. 10.1016/j.maturitas.2019.09.004. 31547918

[100] Steptoe A, Breeze E, Banks J, Nazroo J. Cohort Profile: The English Longitudinal Study of Ageing. Int J Epidemiol. 2013;42(6):1640–8. 10.1093/ije/dys168. PMC3900867 23143611

[101] Banks J, Batty GD, Breedvelt J, Coughlin K, Crawford R, Marmot M et al. ELSA English Longitudinal Study of AgeingEnglish Longitudinal Study of Ageing: Waves 0–11, 1998–2024 [Internet]. UK Data Service; 2025 [cited 2026 Sep 20]. Available from: https://datacatalogue.ukdataservice.ac.uk/studies/study/5050#doi

[102] Ward K, Medina J, Mo M, Cox K. ELSA wave three: life history interview [Internet]. National Centre for Social Research; 2007 [cited 2025 Dec 18]. Available from: https://www.semanticscholar.org/paper/ELSA-wave-three%3A-life-history-interview-Ward-Medina/8561d5a6986e6e6e79d66747b7b5741d3c9304ab

[103] Radloff LS, The CES-D, Scale. A Self-Report Depression Scale for Research in the General Population. Appl Psychol Meas. 1977;1(3):385–401. 10.1177/014662167700100306.

[104] Gallagher D, Kiss A, Lanctot K, Herrmann N. Depressive symptoms and cognitive decline: A longitudinal analysis of potentially modifiable risk factors in community dwelling older adults. J Affect Disord. 2016;190:235–40. 10.1016/j.jad.2015.09.046. 26521086

[105] Veronese N, Solmi M, Maggi S, Noale M, Sergi G, Manzato E, et al. Frailty and incident depression in community-dwelling older people: results from the ELSA study. Int J Geriatr Psychiatry. 2017;32(12). 10.1002/gps.4673. 28195361

[106] Steffick D, Wallace RB, Herzog AR, Ofstedal MB, Fonda S, Langa K. Documentation of Affective Functioning Measures in the Health and Retirement Study [Internet]. Institute for Social Research, University of Michigan; 2000 [cited 2025 Dec 18]. Available from: http://hrsonline.isr.umich.edu/sitedocs/userg/dr-005.pdf

[107] Sexton E, King-Kallimanis BL, Conroy RM, Hickey A. Psychometric evaluation of the CASP-19 quality of life scale in an older Irish cohort. Qual Life Res. 2013;22(9):2549–59. 10.1007/s11136-013-0388-7. 23504522

[108] Wiggins RD, Netuveli G, Hyde M, Higgs P, Blane D. The Evaluation of a Self-enumerated Scale of Quality of Life (CASP-19) in the Context of Research on Ageing: A Combination of Exploratory and Confirmatory Approaches. Soc Indic Res. 2008;89(1):61–77. 10.1007/s11205-007-9220-5.

[109] Sim J, Bartlam B, Bernard M. The CASP-19 as a measure of quality of life in old age: evaluation of its use in a retirement community. Qual Life Res. 2011;20(7):997–1004. 10.1007/s11136-010-9835-x. 21243529

[110] Carstensen TBW, Ørnbøl E, Fink P, Jørgensen T, Dantoft TM, Madsen AL, et al. Adverse life events in the general population - a validation of the cumulative lifetime adversity measure. Eur J Psychotraumatology. 2020;11(1):1717824. 10.1080/20008198.2020.1717824. PMC7034458 32128043

[111] Seery MD, Leo RJ, Lupien SP, Kondrak CL, Almonte JL. An Upside to Adversity? Moderate Cumulative Lifetime Adversity Is Associated With Resilient Responses in the Face of Controlled Stressors. Psychol Sci. 2013;24(7):1181–9. 10.1177/0956797612469210. 23673992

[112] Robins LN, National Institute of Mental Health Diagnostic Interview Schedule. Its History, Characteristics, and Validity. Arch Gen Psychiatry. 1981;38(4):381. 10.1001/archpsyc.1981.01780290015001. 6260053

[113] Holman EA. Traumatic Life Events in Primary Care Patients: A Study in an Ethnically Diverse Sample. Arch Fam Med. 2000;9(9):802–10. 10.1001/archfami.9.9.802. 11031385

[114] Assari S, Lankarani MM. Stressful Life Events and Risk of Depression 25 Years Later: Race and Gender Differences. Front Public Health. 2016;4. 10.3389/fpubh.2016.00049. PMC4805579 27047914

[115] Mamudu L, Li J, McEligot AJ, Wood M, Rusmevichientong P, Tetteh-Bator E, et al. Cancer worry and its impact on self-reported depressive symptoms among adult males and females in the US: a nationwide sample study. BMC Psychiatry. 2024;24(1):31. 10.1186/s12888-023-05405-4. PMC10773041 38191340

[116] Paul KI, Moser K. Unemployment impairs mental health: Meta-analyses. J Vocat Behav. 2009;74(3):264–82. 10.1016/j.jvb.2009.01.001.

[117] Amato PR. The Consequences of Divorce for Adults and Children. J Marriage Fam. 2000;62(4):1269–87. 10.1111/j.1741-3737.2000.01269.x.

[118] Jiang C, Song H, Shi J. The impact of widowhood on mental health of older adults. Geriatr Nur (Lond). 2023;50:38–43. 10.1016/j.gerinurse.2022.12.019. 36640517

[119] Lin I, Brown SL. Consequences of Later-Life Divorce and Widowhood for Adult Well‐Being: A Call for the Convalescence Model. J Fam Theory Rev. 2020;12(2):264–77. 10.1111/jftr.12366. PMC12439811 40964659

[120] Jones TM, Nurius P, Song C, Fleming CM. Modeling life course pathways from adverse childhood experiences to adult mental health. Child Abuse Negl. 2018;80:32–40. 10.1016/j.chiabu.2018.03.005. PMC5953821 29567455

[121] Maroto ML. Pathways into Bankruptcy: Accumulating Disadvantage and the Consequences of Adverse Life Events. Sociol Inq. 2015;85(2):183–216. 10.1111/soin.12073.

[122] Rabaté S, Tô M, van den Berge W, van der Wal W, Tréguier J. The Effect of Adverse Life-Events on Income Trajectories. CPB Discuss Pap. 2025;1–56. 10.34932/74D0-F494.

[123] Krieger N, Fee E. Social Class: The Missing Link in U.S. Health Data. Int J Health Serv. 1994;24(1):25–44. 10.2190/2JG7-YMD5-WCP2-XXNT. 8150566

[124] Walsemann KM, Bell BA, Hummer RA. Effects of Timing and Level of Degree Attained on Depressive Symptoms and Self-Rated Health at Midlife. Am J Public Health. 2012;102(3):557–63. 10.2105/AJPH.2011.300216. PMC3487654 22390521

[125] Austin PC, Hux JE. A brief note on overlapping confidence intervals. J Vasc Surg. 2002;36(1):194–5. 10.1067/mva.2002.125015. 12096281

[126] MacGregor-Fors I, Payton ME. Contrasting Diversity Values: Statistical Inferences Based on Overlapping Confidence Intervals. Quince C, editor. PLoS ONE. 2013;8(2):e56794. 10.1371/journal.pone.0056794 PMC3577692 23437239

[127] R Core Team. R: A Language and Environment for Statistical Computing [Internet]. Vienna, Austria: R Foundation for Statistical Computing. 2026. Available from: https://www.R-project.org/

[128] Agyapong B, Shalaby R, Eboreime E, Obuobi-Donkor G, Owusu E, Adu MK, et al. Cumulative trauma from multiple natural disasters increases mental health burden on residents of Fort McMurray. Eur J Psychotraumatology. 2022;13(1):2059999. 10.1080/20008198.2022.2059999. PMC9116266 35599978

[129] Toffol E, Koponen P, Partonen T. Miscarriage and mental health: Results of two population-based studies. Psychiatry Res. 2013;205(1–2):151–8. 10.1016/j.psychres.2012.08.029. 22985545

[130] Chen S, Chang S, Kuo P, Chen C. Stress, anxiety and depression perceived by couples with recurrent miscarriage. Int J Nurs Pract. 2020;26(2):e12796. 10.1111/ijn.12796. 31713284

[131] Luhmann M, Eid M. Does it really feel the same? Changes in life satisfaction following repeated life events. J Pers Soc Psychol. 2009;97(2):363–81. 10.1037/a0015809. 19634981

[132] Epel ES, Crosswell AD, Mayer SE, Prather AA, Slavich GM, Puterman E, et al. More than a feeling: A unified view of stress measurement for population science. Front Neuroendocrinol. 2018;49:146–69. 10.1016/j.yfrne.2018.03.001. PMC6345505 29551356

[133] Hobson CJ, Kamen J, Szostek J, Nethercut CM, Tiedmann JW, Wojnarowicz S. Stressful Life Events: A Revision and Update of the Social Readjustment Rating Scale. Int J Stress Manag. 1998;5(1):1–23. 10.1023/A:1022978019315.

[134] Cruwys T, Dingle GA, Haslam C, Haslam SA, Jetten J, Morton TA. Social group memberships protect against future depression, alleviate depression symptoms and prevent depression relapse. Soc Sci Med. 2013;98:179–86. 10.1016/j.socscimed.2013.09.013. 24331897

[135] Haslam C, Cruwys T, Haslam SA. The we’s have it: Evidence for the distinctive benefits of group engagement in enhancing cognitive health in aging. Soc Sci Med. 2014;120:57–66. 10.1016/j.socscimed.2014.08.037. 25222136

[136] Rafnsson SB, Shankar A, Steptoe A. Longitudinal Influences of Social Network Characteristics on Subjective Well-Being of Older Adults: Findings From the ELSA Study. J Aging Health. 2015;27(5):919–34. 10.1177/0898264315572111. 25804898

[137] Rollings J, Micheletta J, Van Laar D, Waller BM. Personality Traits Predict Social Network Size in Older Adults. Pers Soc Psychol Bull. 2023;49(6):925–38. 10.1177/01461672221078664. PMC10226003 35393911

[138] White J, Zaninotto P, Walters K, Kivimäki M, Demakakos P, Biddulph J, et al. Duration of depressive symptoms and mortality risk: The English Longitudinal Study of Ageing (ELSA). Br J Psychiatry. 2016;208(4):337–42. 10.1192/bjp.bp.114.155333. PMC4816969 26795425

[139] Borrat-Besson C, Ryser V-A, Gonçalves J. An evaluation of the CASP-12 scale used in the Survey of Health, Ageing and Retirement in Europe (SHARE) to measure Quality of Life among people aged 50+. FORS Working Paper Series. 2015;paper:2015-4. 10.24440/FWP-2015-00004.

[140] Green BL, Goodman LA, Krupnick JL, Corcoran CB, Petty RM, Stockton P, et al. Outcomes of single versus multiple trauma exposure in a screening sample. J Trauma Stress. 2000;13(2):271–86. 10.1023/A:1007758711939. 10838675

[141] Miller MA, Rahe RH. Life changes scaling for the 1990s. J Psychosom Res. 1997;43(3):279–92. 10.1016/S0022-3999(97)00118-9. 9304554

[142] Austenfeld JL, Stanton AL. Coping Through Emotional Approach: A New Look at Emotion, Coping, and Health-Related Outcomes. J Pers. 2004;72(6):1335–64. 10.1111/j.1467-6494.2004.00299.x. 15509285

[143] Blane DB. Collecting retrospective data: Development of a reliable method and a pilot study of its use. Soc Sci Med. 1996;42(5):751–7. 10.1016/0277-9536(95)00340-1. 8685743

[144] Hardt J, Rutter M. Validity of adult retrospective reports of adverse childhood experiences: review of the evidence. J Child Psychol Psychiatry. 2004;45(2):260–73. 10.1111/j.1469-7610.2004.00218.x. 14982240

[145] Bloise SM, Johnson MK. Memory for emotional and neutral information: Gender and individual differences in emotional sensitivity. Memory. 2007;15(2):192–204. 10.1080/09658210701204456. 17534112

[146] Boals A. Events that have become central to identity: Gender differences in the centrality of events scale for positive and negative events. Appl Cogn Psychol. 2010;24(1):107–21. 10.1002/acp.1548.

[147] Davis PJ. Gender differences in autobiographical memory for childhood emotional experiences. J Pers Soc Psychol. 1999;76(3):498–510. 10.1037/0022-3514.76.3.498. 10101879

[148] Davis MC, Matthews KA, Twamley EW. Is life more difficult on mars or venus? A meta-analytic review of sex differences in major and minor life events. Ann Behav Med. 1999;21(1):83–97. 10.1007/BF02895038. 18425659

[149] Stratton E, Einboden R, Ryan R, Choi I, Harvey SB, Glozier N. Deciding to Disclose a Mental Health Condition in Male Dominated Workplaces; A Focus-Group Study. Front Psychiatry. 2018;9. 10.3389/fpsyt.2018.00684. PMC6305748 30618865

[150] Bogner HR, Gallo JJ. Are higher rates of depression in women accounted for by differential symptom reporting? Soc Psychiatry Psychiatr Epidemiol. 2004;39(2):126–32. 10.1007/s00127-004-0714-. z PubMed PMID: 15052394; PubMed Central PMCID: PMC2830739. PMC2830739 15052394

[151] Acciai F, Hardy M. Depression in later life: A closer look at the gender gap. Soc Sci Res. 2017;68:163–75. 10.1016/j.ssresearch.2017.08.003. 29108595

[152] Canadian Institutes of Health Research. Sex and Gender in Health Research [Internet]. 2018 [cited 2025 Dec 19]. Sex and Gender in Health Research – CIHR. Available from: https://cihr-irsc.gc.ca/e/50833.html

[153] Mann CJ. Observational research methods. Research design II: cohort, cross sectional, and case-control studies. Emerg Med J. 2003;20(1):54–60. 10.1136/emj.20.1.54. PMC1726024 12533370

[154] Phillips AC, Carroll D, Der G. Negative life events and symptoms of depression and anxiety: stress causation and/or stress generation. Anxiety Stress Coping. 2015;28(4):357–71. 10.1080/10615806.2015.1005078. PMC4772121 25572915

[155] Pescosolido BA, Halpern-Manners A, Luo L, Perry B. Trends in Public Stigma of Mental Illness in the US, 1996–2018. JAMA Netw Open. 2021;4(12):e2140202. 10.1001/jamanetworkopen.2021.40202. PMC8693212 34932103

[156] Eagly AH, Nater C, Miller DI, Kaufmann M, Sczesny S. Gender stereotypes have changed: A cross-temporal meta-analysis of U.S. public opinion polls from 1946 to 2018. Am Psychol. 2020;75(3):301–15. 10.1037/amp0000494. 31318237

[157] Meagher KD, Shu X. Trends in U.S. Gender Attitudes, 1977 to 2018: Gender and Educational Disparities. Socius Sociol Res Dyn World. 2019;5:2378023119851692. 10.1177/2378023119851692.

[158] Bloom DE, Canning D, Mansfield RK, Moore M. Demographic change, social security systems, and savings. J Monet Econ. 2007;54(1):92–114. 10.1016/j.jmoneco.2006.12.004. PMC2768304 19865594

[159] Durrani H. Healthcare and healthcare systems: inspiring progress and future prospects. mHealth. 2016;2:3. 10.3978/j.issn.2306-9740. 2016.01.03 PubMed PMID: 28293581; PubMed Central PMCID: PMC5344175. PMC5344175 28293581

[160] Kleinau E, Schmid R. Occupation Children’ in Germany after World War II — Problems and Coping Strategies. Child Soc. 2019;33(3):239–52. 10.1111/chso.12323.

[161] Rodríguez-Martínez A, De-la-Fuente-Robles YM, Martín-Cano MDC, Jiménez-Delgado JJ. Quality of Life and Well-Being of Older Adults in Nursing Homes. Syst Rev Soc Sci. 2023;12(7):418. 10.3390/socsci12070418.

