# Supplementary material for: The accumulation of adverse life events across the life course and their association with mental health and well-being: exploring gender heterogeneity
Source: BMC Public Health. 2026 Oct 23;26:3054. doi: 10.1186/s12889-026-30076-7 (PMC13693152; doi:10.1186/s12889-026-30076-7)
Supplement: Supplementary file 1 [file 12889_2026_30076_MOESM1_ESM.docx]

**Additional File 1**

**Title: Comparison of included vs. excluded participants.**

| Table A1: Differences in key characteristics between included and excluded individuals to assess potential selection bias. | | | |
| --- | --- | --- | --- |
|  | Included  (n = 5064) | Excluded^a^  (n = 4707) | p-value^b^ |
| Gender |  |  | 0.821 |
| Men | 2232 (44.1) | 2063 (43.8) |  |
| Women | 2832 (55.9) | 2644 (56.2) |  |
| Age |  |  | <0.001 |
| Mean (SD) | 64.53 (10.27) | 66.24 (12.02) |  |
| Birthplace |  |  | <0.001 |
| UK | 4781 (94.4) | 4225 (90.0) |  |
| Outside of UK | 283 (5.6) | 468 (10.0) |  |
| Highest education |  |  | <0.001 |
| No qualification | 1185 (23.4) | 1630 (34.9) |  |
| Low education | 1615 (31.9) | 1365 (29.3) |  |
| Middle education | 1243 (24.5) | 990 (21.2) |  |
| High education | 1021 (20.2) | 679 (14.6) |  |
| HH-equivalent income |  |  | <0.001 |
| Mean (SD) | 328.26 (246.65) | 293.76 (263.32) |  |
| Labour force status |  |  | <0.001 |
| In labour force | 1952 (38.6) | 1736 (37.0) |  |
| Unemployed | 667 (13.2) | 773 (16.5) |  |
| Retired | 2438 (48.2) | 2179 (46.5) |  |
| Partnership status |  |  | <0.001 |
| Married | 3555 (70.2) | 2836 (60.3) |  |
| Cohabiting | 266 (5.3) | 242 (5.1) |  |
| Neither | 1243 (24.5) | 1629 (34.6) |  |
| Num. HH children |  |  | <0.001 |
| Mean (SD) | 0.34 (0.71) | 0.39 (0.78) |  |
| Social participation |  |  | <0.001 |
| Mean (SD) | 1.55 (1.38) | 1.34 (1.32) |  |
| Social network size |  |  | 0.237 |
| Mean (SD) | 7.72 (4.15) | 7.60 (4.21) |  |
| *Notes:* Absolute numbers (percentages) are presented for nominal and ordinal variables; mean values (standard deviation) are presented for continuous variables.  ^a^ Included individuals have complete data for all characteristics shown; for excluded individuals, descriptive statistics are based on available data only.  ^b^ *p*-values indicate differences between included and excluded individuals based on chi-square tests for categorical variables and *t*-tests for continuous variables.  Abbreviations: SD = standard deviation; UK = United Kingdom; HH = household. | | | |
