# Supplementary material for: The accumulation of adverse life events across the life course and their association with mental health and well-being: exploring gender heterogeneity
Source: BMC Public Health. 2026 Oct 23;26:3054. doi: 10.1186/s12889-026-30076-7 (PMC13693152; doi:10.1186/s12889-026-30076-7)
Supplement: Supplementary file 2 [file 12889_2026_30076_MOESM2_ESM.docx]

**Additional File 2**

**Title: Testing for non-linear effects of cumulative adverse life events on mental health and well-being.**

| Table A1: Regression analyses testing quadratic associations between TCALE and all outcomes. | | | |
| --- | --- | --- | --- |
|  | Depressive symptomatology  OR [95% CI] | Life satisfaction  β [95% CI] | Quality of life  β [95% CI] |
| TCALE | 1.39 *** [1.21; 1.61] | -0.68 *** [-0.94; -0.43] | -0.72 *** [-1.05; -0.40] |
| TCALE ^ 2 | 0.99 [0.98; 1.01] | -0.01 [-0.04; 0.02] | -0.02 [-0.06; 0.01] |
| Gender: Men (ref.) | - | - | - |
| Women | 1.67 *** [1.38; 2.02] | -0.38 * [-0.70; -0.05] | 0.53 * [0.11; 0.96] |
| Age: ≤ 59 years (ref.) | - | - | - |
| 60-69 years | 0.86 [0.69; 1.08] | 0.57 * [0.13; 1.02] | 0.55 [-0.02; 1.12] |
| 70-79 years | 0.75 * [0.59; 0.96] | 1.58 *** [1.12; 2.05] | 0.21 [-0.41; 0.83] |
| 80+ years | 0.89 [0.64; 1.25] | 1.28 *** [0.61; 1.94] | -2.17 *** [-3.06; -1.28] |
| Birthplace: UK (ref.) | - | - | - |
| Outside of UK | 1.62 ** [1.14; 2.30] | 0.23 [-0.52; 0.99] | -0.59 [-1.57; 0.39] |
| Highest education: No qualification (ref.) | - | - | - |
| Low education | 0.59 *** [0.48; 0.74] | -0.18 [-0.64; 0.28] | 1.43 *** [0.80; 2.05] |
| Medium education | 0.48 *** [0.37; 0.61] | 0.38 [-0.13; 0.89] | 2.69 *** [2.01; 3.38] |
| High education | 0.27 *** [0.20; 0.37] | 0.87 ** [0.32; 1.41] | 3.86 *** [3.14; 4.57] |
| Intercept | 0.05 *** [0.03; 0.08] | 22.17 *** [21.45; 22.89] | 42.53 *** [41.60; 43.46] |
| *Notes:* Shown values are odds ratios or beta coefficients, with 95% confidence intervals in brackets. Standard errors are clustered at the household level.  Significance levels: < 0.05*, < 0.01**, < 0.001***.  Abbreviations: OR = odds ratio; β = beta coefficient; 95% CI = 95% confidence interval; ref. = reference category; TCALE = total cumulative adverse life event score; UK = United Kingdom. | | | |

| Table A2: Regression analyses testing quadratic associations between SOCALE and OOCALE and all outcomes. | | | |
| --- | --- | --- | --- |
|  | Depressive symptomatology  OR [95% CI] | Life satisfaction  β [95% CI] | Quality of life  β [95% CI] |
| SOALE | 1.35 *** [1.14; 1.59] | -0.82 *** [-1.12; -0.51] | -1.13 *** [-1.50; -0.75] |
| SOALE ^ 2 | 1.00 [0.98; 1.03] | -0.03 [-0.08; 0.02] | -0.02 [-0.09; 0.04] |
| OOALE | 1.26 * [1.04; 1.54] | -0.51 ** [-0.89; -0.13] | -0.40 [-0.92; 0.12] |
| OOALE ^ 2 | 0.99 [0.95; 1.03] | 0.02 [-0.06; 0.11] | -0.00 [-0.12; 0.12] |
| Gender: Men (ref.) | - | - | - |
| Women | 1.66 *** [1.37; 2.01] | -0.36 * [-0.68; -0.03] | 0.55 * [0.12; 0.97] |
| Age: ≤ 59 years (ref.) | - | - | - |
| 60-69 years | 0.83 [0.66; 1.04] | 0.72 ** [0.27; 1.17] | 0.77 ** [0.19; 1.34] |
| 70-79 years | 0.73 * [0.57; 0.93] | 1.69 *** [1.23; 2.16] | 0.38 [-0.24; 1.00] |
| 80+ years | 0.93 [0.67; 1.29] | 1.19 *** [0.53; 1.85] | -2.27 *** [-3.16; -1.38] |
| Birthplace: UK (ref.) | - | - | - |
| Outside of UK | 1.66 ** [1.17; 2.36] | 0.13 [-0.61; 0.88] | -0.74 [-1.71; 0.23] |
| Highest education: No qualification (ref.) | - | - | - |
| Low education | 0.60 *** [0.48; 0.74] | -0.20 [-0.66; 0.26] | 1.39 *** [0.77; 2.02] |
| Medium education | 0.48 *** [0.37; 0.62] | 0.32 [-0.19; 0.83] | 2.59 *** [1.91; 3.28] |
| High education | 0.27 *** [0.19; 0.37] | 0.83 ** [0.28; 1.38] | 3.78 *** [3.06; 4.49] |
| Intercept | 0.06 *** [0.04; 0.09] | 22.09 *** [21.40; 22.77] | 42.57 *** [41.68; 43.46] |
| *Notes:* Shown values are odds ratios or beta coefficients, with 95% confidence intervals in brackets. Standard errors are clustered at the household level.  Significance levels: < 0.05*, < 0.01**, < 0.001***.  Abbreviations: OR = odds ratio; β = beta coefficient; 95% CI = 95% confidence interval; ref. = reference category; SOCALE = self-oriented cumulative adverse life event score; OOCALE = other-oriented cumulative adverse life event score; UK = United Kingdom. | | | |

*Figure A1: Smoothing spline estimates of adverse life event scores on considered outcomes.*

*Notes: Lines represent penalised smoothing spline estimates of the marginal association between adverse life event scores and depressive symptomatology, life satisfaction, and quality of life, with 95% confidence intervals. Predicted outcomes represent estimates averaged over the observed distribution of all covariates. Standard errors are clustered at the household level. The dashed vertical line separates the model including TCALE from the model including SOCALE and OOCALE as predictors.*

*Abbreviations: TCALE = Total cumulative adverse life event score; SOCALE = Self-oriented cumulative adverse life event score; OOCALE = Other-oriented cumulative adverse life event score.*
