# Supplementary material for: The accumulation of adverse life events across the life course and their association with mental health and well-being: exploring gender heterogeneity
Source: BMC Public Health. 2026 Oct 23;26:3054. doi: 10.1186/s12889-026-30076-7 (PMC13693152; doi:10.1186/s12889-026-30076-7)
Supplement: Supplementary file 3 [file 12889_2026_30076_MOESM3_ESM.docx]

**Additional File 3**

**Title: Gender-stratified regression models.**

| Table A1: Logistic regression models predicting depressive symptomatology, stratified by gender. | | | | |
| --- | --- | --- | --- | --- |
|  | **Men** | | **Women** | |
| TCALE | 1.24 *** [1.17; 1.32] | - | 1.34 *** [1.27; 1.41] | - |
| SOCALE | - | 1.30 *** [1.18; 1.43] | - | 1.41 *** [1.31; 1.51] |
| OOCALE | - | 1.17 ** [1.04; 1.31] | - | 1.23 *** [1.11; 1.36] |
| Age: ≤ 59 years (ref.) | - | - | - | - |
| 60-69 years | 0.98 [0.68; 1.42] | 0.95 [0.65; 1.38] | 0.80 [0.61; 1.06] | 0.77 [0.58; 1.02] |
| 70-79 years | 0.62 * [0.41; 0.92] | 0.59 * [0.39; 0.90] | 0.84 [0.62; 1.14] | 0.83 [0.61; 1.12] |
| 80+ years | 0.83 [0.47; 1.46] | 0.83 [0.47; 1.48] | 0.96 [0.64; 1.44] | 0.99 [0.66; 1.49] |
| Birthplace: UK (ref.) | - | - | - | - |
| Outside of UK | 1.58 [0.84; 2.97] | 1.61 [0.86; 3.03] | 1.65 * [1.06; 2.56] | 1.70 * [1.10; 2.64] |
| Highest education: No qualification (ref.) | - | - | - | - |
| Low education | 0.57 ** [0.39; 0.85] | 0.58 ** [0.39; 0.86] | 0.61 *** [0.46; 0.80] | 0.61 *** [0.46; 0.80] |
| Medium education | 0.38 *** [0.25; 0.57] | 0.39 *** [0.26; 0.58] | 0.55 *** [0.40; 0.76] | 0.55 *** [0.40; 0.76] |
| High education | 0.24 *** [0.15; 0.39] | 0.25 *** [0.15; 0.40] | 0.28 *** [0.18; 0.43] | 0.28 *** [0.18; 0.43] |
| Intercept | 0.08 *** [0.05; 0.14] | 0.09 *** [0.05; 0.14] | 0.09 *** [0.06; 0.12] | 0.09 *** [0.06; 0.13] |
| *Notes:* Shown values are odds ratios, with 95% confidence intervals in brackets. Standard errors are clustered at the household level.  Significance levels: < 0.05*, < 0.01**, < 0.001***.  Abbreviations: ref. = reference category; TCALE = total cumulative adverse life event score; SOCALE = self-oriented cumulative adverse life event score; OOCALE = other-oriented cumulative adverse life event score; UK = United Kingdom. | | | | |

| Table A2: Linear regression models predicting life satisfaction, stratified by gender. | | | | |
| --- | --- | --- | --- | --- |
|  | **Men** | | **Women** | |
| TCALE | -0.64 *** [-0.75; -0.52] | - | -0.88 *** [-1.00; -0.76] | - |
| SOCALE | - | -0.64 *** [-0.77; -0.51] | - | -0.88 *** [-1.00; -0.76] |
| OOCALE | - | - | -0.93 *** [-1.12; -0.74] | - |
| Age: ≤ 59 years (ref.) | - | - | - | - |
| 60-69 years | 0.65 [-0.00; 1.31] | 0.85 * [0.19; 1.51] | 0.54 [-0.04; 1.12] | 0.65 * [0.07; 1.23] |
| 70-79 years | 1.89 *** [1.22; 2.56] | 2.09 *** [1.41; 2.77] | 1.33 *** [0.74; 1.93] | 1.40 *** [0.80; 2.00] |
| 80+ years | 2.33 *** [1.41; 3.26] | 2.29 *** [1.37; 3.22] | 0.43 [-0.44; 1.30] | 0.36 [-0.51; 1.22] |
| Birthplace: UK (ref.) | - | - | - | - |
| Outside of UK | -0.08 [-1.19; 1.02] | -0.19 [-1.30; 0.92] | 0.33 [-0.64; 1.30] | 0.25 [-0.71; 1.22] |
| Highest education: No qualification (ref.) | - | - | - | - |
| Low education | 0.02 [-0.76; 0.80] | -0.07 [-0.85; 0.71] | -0.35 [-0.94; 0.23] | -0.35 [-0.94; 0.23] |
| Medium education | 0.37 [-0.42; 1.17] | 0.20 [-0.60; 1.00] | 0.39 [-0.28; 1.05] | 0.36 [-0.30; 1.03] |
| High education | 0.74 [-0.05; 1.52] | 0.58 [-0.20; 1.37] | 1.04 ** [0.28; 1.81] | 1.03 ** [0.27; 1.79] |
| Intercept | 21.64 *** [20.77; 22.52] | 21.63 *** [20.76; 22.50] | 22.53 *** [21.82; 23.25] | 22.39 *** [21.67; 23.11] |
| *Notes:* Shown values are beta coefficients, with 95% confidence intervals in brackets. Standard errors are clustered at the household level.  Significance levels: < 0.05*, < 0.01**, < 0.001***.  Abbreviations: ref. = reference category; TCALE = total cumulative adverse life event score; SOCALE = self-oriented cumulative adverse life event score; OOCALE = other-oriented cumulative adverse life event score; UK = United Kingdom. | | | | |

| Table A3: Linear regression models predicting quality of life, stratified by gender. | | | | |
| --- | --- | --- | --- | --- |
|  | **Men** | | **Women** | |
| TCALE | -0.79 *** [-0.97; -0.62] | - | -1.04 *** [-1.20; -0.88] | - |
| SOCALE | - | -1.21 *** [-1.46; -0.97] | - | -1.31 *** [-1.52; -1.10] |
| OOCALE | - | -0.24 [-0.54; 0.06] | - | -0.58 *** [-0.87; -0.29] |
| Age: ≤ 59 years (ref.) | - | - | - | - |
| 60-69 years | 0.60 [-0.24; 1.45] | 0.88 * [0.04; 1.73] | 0.53 [-0.20; 1.26] | 0.71 [-0.02; 1.45] |
| 70-79 years | 0.73 [-0.17; 1.64] | 1.02 * [0.11; 1.94] | -0.25 [-1.05; 0.56] | -0.13 [-0.94; 0.67] |
| 80+ years | -1.22 [-2.50; 0.06] | -1.28 * [-2.55; -0.00] | -2.92 *** [-4.07; -1.76] | -3.04 *** [-4.18; -1.89] |
| Birthplace: UK (ref.) | - | - | - | - |
| Outside of UK | -0.77 [-2.26; 0.72] | -0.92 [-2.42; 0.57] | -0.60 [-1.86; 0.65] | -0.73 [-1.98; 0.51] |
| Highest education: No qualification (ref.) | - | - | - | - |
| Low education | 1.69 ** [0.63; 2.75] | 1.56 ** [0.50; 2.61] | 1.21 ** [0.42; 2.00] | 1.21 ** [0.43; 2.00] |
| Medium education | 2.65 *** [1.59; 3.70] | 2.40 *** [1.34; 3.46] | 2.78 *** [1.90; 3.67] | 2.75 *** [1.87; 3.63] |
| High education | 3.96 *** [2.92; 4.99] | 3.73 *** [2.69; 4.78] | 3.79 *** [2.82; 4.76] | 3.77 *** [2.80; 4.73] |
| Intercept | 42.10 *** [40.92; 43.27] | 42.08 *** [40.91; 43.25] | 44.09 *** [43.15; 45.04] | 43.86 *** [42.91; 44.82] |
| *Notes:* Shown values are beta coefficients, with 95% confidence intervals in brackets. Standard errors are clustered at the household level.  Significance levels: < 0.05*, < 0.01**, < 0.001***.  Abbreviations: ref. = reference category; TCALE = total cumulative adverse life event score; SOCALE = self-oriented cumulative adverse life event score; OOCALE = other-oriented cumulative adverse life event score; UK = United Kingdom. | | | | |
