# Supplementary material for: The accumulation of adverse life events across the life course and their association with mental health and well-being: exploring gender heterogeneity
Source: BMC Public Health. 2026 Oct 23;26:3054. doi: 10.1186/s12889-026-30076-7 (PMC13693152; doi:10.1186/s12889-026-30076-7)
Supplement: Supplementary file 4 [file 12889_2026_30076_MOESM4_ESM.docx]

**Additional File 4**

**Title: Weights assigned to individual adverse life events for sensitivity analysis.**

| Table A1: Assignment of weights to individual adverse life events. | | |
| --- | --- | --- |
| Adverse life events in ELSA | Event in Hobson et al. (1988)^a^ | Weight |
| Death of long-term partner/spouse | Death of spouse/mate | 87 |
| Death of child | Death of a close family member | 79 |
| Divorce | Divorce | 71 |
| Separation from a long-term or/and cohabiting partner | Seperation or reconciliation with spouse/mate | 66 |
| Diagnosis of a serious physical disease | Major injury/illness to self | 78 |
| Job loss due to company closing | Being fired/laid-off/unemployed | 64 |
| Experienced parental divorce before age 16 | Divorce | 71 |
| Experienced violence between parents before age 16 | Experienced domestic violence/abuse | 69 |
| Witnessed serious injury or death in war or military action | Surviving a disaster | 59 |
| Witnessed a fatal or violent accident (not war) | Surviving a disaster | 59 |
| Lost a very close friend or relative in war or military service | Death of close family member & Death of close friend | 70^b^ |
| Close friend or relative was at risk of death or died due to illness or serious accident | Death of close friend | 61 |
| Death of father | Death of a close family member | 79 |
| Death of mother | Death of a close family member | 79 |
| Experienced a natural disaster | Surviving a disaster | 59 |
| Major injury/illness to self | Major injury/illness to self | 78 |
| Victim of a serious physical attack or assault | Experienced domestic violence/abuse | 69 |
| Victim of sexual assault | Experienced domestic violence/abuse | 69 |
| Fired a weapon or came under fire during combat | Surviving a disaster | 59 |
| Experienced severe financial hardship | Experiencing financial problems/difficulties | 62 |
| Physically abused by parents before age 16 | Experienced domestic violence/abuse | 69 |
| Experienced evacuation during war | Surviving a disaster | 59 |
| ^a^ Hobson CJ, Kamen J, Szostek J, Nethercut CM, Tiedmann JW, Wojnarowicz S. Stressful Life Events: A Revision and Update of the Social Readjustment Rating Scale. Int J Stress Manag. 1998;5(1):1–23.  ^b^ This weight represents the average of the values for “Death of close family member” and “Death of close friend”, as both are covered by the ELSA variable (calculation: (79 + 61) / 2).  Abbreviations: ELSA = English Longitudinal Study of Ageing. | | |
