# Supplementary material for: The accumulation of adverse life events across the life course and their association with mental health and well-being: exploring gender heterogeneity
Source: BMC Public Health. 2026 Oct 23;26:3054. doi: 10.1186/s12889-026-30076-7 (PMC13693152; doi:10.1186/s12889-026-30076-7)
Supplement: Supplementary file 5 [file 12889_2026_30076_MOESM5_ESM.docx]

**Additional File 5**

**Title: Results of sensitivity analyses.**

Four sensitivity analyses were performed, of which the results are presented in this document in the following order:

Sensitivity analysis 1: Accounting for multiple occurrences of six adverse life events in the accumulation score.

Sensitivity analysis 2: Alternative operationalization of the accumulation score, with weighting of adverse life events.

Sensitivity analysis 3: Inclusion of additional confounders in regression models.

Sensitivity analysis 4: Alternative operationalization of outcomes (depression cut-off ≥3; quality of life CASP-12)

**Sensitivity analysis 1:**

| Table A1: Descriptive distribution of experienced adverse life events and accumulation scores, stratified by gender. | | | |
| --- | --- | --- | --- |
|  | Men  (n = 2232) | Women  (n = 2832) | p-value^a^ |
| **Adverse life events** |  |  |  |
| Divorce | 0.24 (0.49) | 0.26 (0.50) | 0.132 |
| Separation from a long-term or/and cohabiting partner | 0.26 (0.68) | 0.31 (0.68) | 0.011 |
| Diagnosis of a serious physical disease | 0.90 (1.06) | 0.99 (1.08) | 0.005 |
| Job loss due to company closing | 0.33 (0.64) | 0.21 (0.50) | <0.001 |
| Death of long-term partner/spouse | 0.10 (0.32) | 0.22 (0.44) | <0.001 |
| Death of child | 0.06 (0.25) | 0.07 (0.29) | 0.047 |
| **Accumulation scores** |  |  |  |
| TCALE | 3.84 (2.21) | 3.57 (1.99) | <0.001 |
| SOCALE | 2.16 (1.50) | 2.00 (1.48) | <0.001 |
| OOCALE | 1.68 (1.27) | 1.57 (1.09) | 0.001 |
| *Notes:* Mean values (standard deviation) are presented.  ^a^ p-values indicating gender differences from two-tailed t-tests.  Abbreviations: TCALE = total cumulative adverse life event score; SOCALE = self-oriented cumulative adverse life event score; OOCALE = other-oriented cumulative adverse life event score. | | | |

| Table A2: Logistic regression models predicting depressive symptomatology. | | | | |
| --- | --- | --- | --- | --- |
|  | **Model 1** | **Model 2** | **Model 3** | **Model 4** |
| TCALE | 1.26 *** [1.22; 1.30] | - | 1.22 *** [1.16; 1.29] | - |
| SOCALE | - | 1.29 *** [1.24; 1.34] | - | 1.25 *** [1.17; 1.34] |
| OOCALE | - | 1.19 *** [1.10; 1.28] | - | 1.15 * [1.03; 1.29] |
| TCALE x Gender | - | - | 1.05 [0.99; 1.13] | - |
| SOCALE x Gender | - | - | - | 1.05 [0.96; 1.14] |
| OOCALE x Gender | - | - | - | 1.05 [0.91; 1.22] |
| Gender: Men (ref.) | - | - | - | - |
| Women | 1.66 *** [1.37; 2.01] | 1.65 *** [1.36; 2.00] | 1.24 [0.83; 1.86] | 1.26 [0.84; 1.89] |
| Age: ≤ 59 years (ref.) | - | - | - | - |
| 60-69 years | 0.81 [0.65; 1.02] | 0.79 * [0.63; 0.99] | 0.81 [0.65; 1.01] | 0.78 * [0.62; 0.99] |
| 70-79 years | 0.68 ** [0.53; 0.87] | 0.67 ** [0.52; 0.86] | 0.68 ** [0.53; 0.87] | 0.67 ** [0.52; 0.86] |
| 80+ years | 0.81 [0.58; 1.13] | 0.82 [0.59; 1.15] | 0.81 [0.58; 1.13] | 0.82 [0.59; 1.15] |
| Birthplace: UK (ref.) | - | - | - | - |
| Outside of UK | 1.67 ** [1.17; 2.38] | 1.71 ** [1.20; 2.44] | 1.68 ** [1.18; 2.40] | 1.72 ** [1.21; 2.45] |
| Highest education: No qualification (ref.) | - | - | - | - |
| Low education | 0.61 *** [0.49; 0.76] | 0.61 *** [0.49; 0.77] | 0.61 *** [0.49; 0.76] | 0.61 *** [0.49; 0.77] |
| Medium education | 0.48 *** [0.38; 0.63] | 0.49 *** [0.38; 0.63] | 0.48 *** [0.37; 0.62] | 0.49 *** [0.38; 0.63] |
| High education | 0.27 *** [0.20; 0.38] | 0.27 *** [0.20; 0.38] | 0.27 *** [0.19; 0.37] | 0.27 *** [0.20; 0.38] |
| Intercept | 0.06 *** [0.05; 0.08] | 0.07 *** [0.05; 0.09] | 0.07 *** [0.05; 0.11] | 0.08 *** [0.05; 0.11] |
| *Notes:* Shown values are odds ratios, with 95% confidence intervals in brackets. Standard errors are clustered at the household level.  Significance levels: < 0.05*, < 0.01**, < 0.001***.  Abbreviations: ref. = reference category; TCALE = total cumulative adverse life event score; SOCALE = self-oriented cumulative adverse life event score; OOCALE = other-oriented cumulative adverse life event score; UK = United Kingdom. | | | | |

| Table A3: Linear regression models predicting life satisfaction. | | | | |
| --- | --- | --- | --- | --- |
|  | **Model 1** | **Model 2** | **Model 3** | **Model 4** |
| TCALE | -0.67 *** [-0.75; -0.60] | - | -0.61 *** [-0.72; -0.50] | - |
| SOCALE | - | -0.79 *** [-0.89; -0.69] | - | -0.78 *** [-0.92; -0.64] |
| OOCALE | - | -0.40 *** [-0.55; -0.24] | - | -0.24 * [-0.45; -0.03] |
| TCALE x Gender | - | - | -0.13 [-0.27; 0.01] | - |
| SOCALE x Gender | - | - | - | -0.02 [-0.20; 0.17] |
| OOCALE x Gender | - | - | - | -0.32 * [-0.61; -0.03] |
| Gender: Men (ref.) | - | - | - | - |
| Women | -0.35 * [-0.67; -0.03] | -0.34 * [-0.66; -0.02] | 0.19 [-0.43; 0.81] | 0.23 [-0.40; 0.86] |
| Age: ≤ 59 years (ref.) | - | - | - | - |
| 60-69 years | 0.69 ** [0.25; 1.14] | 0.82 *** [0.37; 1.27] | 0.70 ** [0.25; 1.14] | 0.82 *** [0.37; 1.27] |
| 70-79 years | 1.76 *** [1.30; 2.22] | 1.87 *** [1.40; 2.33] | 1.76 *** [1.30; 2.22] | 1.87 *** [1.40; 2.33] |
| 80+ years | 1.53 *** [0.88; 2.19] | 1.50 *** [0.84; 2.15] | 1.53 *** [0.88; 2.19] | 1.50 *** [0.84; 2.15] |
| Birthplace: UK (ref.) | - | - | - | - |
| Outside of UK | 0.15 [-0.61; 0.90] | 0.07 [-0.68; 0.82] | 0.14 [-0.62; 0.89] | 0.06 [-0.69; 0.81] |
| Highest education: No qualification (ref.) | - | - | - | - |
| Low education | -0.25 [-0.71; 0.21] | -0.29 [-0.74; 0.17] | -0.25 [-0.71; 0.20] | -0.30 [-0.75; 0.16] |
| Medium education | 0.30 [-0.21; 0.80] | 0.22 [-0.28; 0.73] | 0.30 [-0.21; 0.81] | 0.21 [-0.30; 0.72] |
| High education | 0.76 ** [0.22; 1.31] | 0.71 * [0.17; 1.25] | 0.78 ** [0.23; 1.32] | 0.71 * [0.16; 1.25] |
| Intercept | 22.31 *** [21.71; 22.91] | 22.14 *** [21.54; 22.74] | 22.02 *** [21.35; 22.68] | 21.85 *** [21.19; 22.52] |
| *Notes***:** Shown values are beta coefficients, with 95% confidence intervals in brackets. Standard errors are clustered at the household level.  Significance levels: < 0.05*, < 0.01**, < 0.001***.  Abbreviations: ref. = reference category; TCALE = total cumulative adverse life event score; SOCALE = self-oriented cumulative adverse life event score; OOCALE = other-oriented cumulative adverse life event score; UK = United Kingdom. | | | | |

| Table A4: Linear regression models predicting quality of life. | | | | |
| --- | --- | --- | --- | --- |
|  | **Model 1** | **Model 2** | **Model 3** | **Model 4** |
| TCALE | -0.87 *** [-0.96; -0.77] | - | -0.80 *** [-0.94; -0.66] | - |
| SOCALE | - | -1.08 *** [-1.20; -0.96] | - | -1.07 *** [-1.25; -0.88] |
| OOCALE | - | -0.36 *** [-0.56; -0.15] | - | -0.22 [-0.51; 0.06] |
| TCALE x Gender | - | - | -0.14 [-0.32; 0.05] | - |
| SOCALE x Gender | - | - | - | -0.03 [-0.27; 0.21] |
| OOCALE x Gender | - | - | - | -0.27 [-0.66; 0.12] |
| Gender: Men (ref.) | - | - | - | - |
| Women | 0.56 ** [0.14; 0.98] | 0.58 ** [0.16; 1.00] | 1.15 ** [0.32; 1.98] | 1.10 * [0.25; 1.94] |
| Age: ≤ 59 years (ref.) | - | - | - | - |
| 60-69 years | 0.69 * [0.13; 1.26] | 0.93 ** [0.36; 1.49] | 0.70 * [0.13; 1.26] | 0.92 ** [0.36; 1.49] |
| 70-79 years | 0.44 [-0.18; 1.06] | 0.64 * [0.02; 1.26] | 0.44 [-0.18; 1.06] | 0.64 * [0.02; 1.26] |
| 80+ years | -1.80 *** [-2.68; -0.92] | -1.87 *** [-2.75; -1.00] | -1.80 *** [-2.68; -0.92] | -1.87 *** [-2.75; -1.00] |
| Birthplace: UK (ref.) | - | - | - | - |
| Outside of UK | -0.70 [-1.68; 0.27] | -0.86 [-1.83; 0.11] | -0.72 [-1.69; 0.26] | -0.86 [-1.83; 0.11] |
| Highest education: No qualification (ref.) | - | - | - | - |
| Low education | 1.34 *** [0.72; 1.96] | 1.27 *** [0.66; 1.89] | 1.33 *** [0.72; 1.95] | 1.27 *** [0.65; 1.88] |
| Medium education | 2.60 *** [1.93; 3.27] | 2.47 *** [1.79; 3.14] | 2.60 *** [1.93; 3.28] | 2.46 *** [1.78; 3.13] |
| High education | 3.72 *** [3.01; 4.43] | 3.62 *** [2.91; 4.32] | 3.73 *** [3.03; 4.44] | 3.62 *** [2.91; 4.33] |
| Intercept | 43.10 *** [42.31; 43.89] | 42.78 *** [41.99; 43.57] | 42.78 *** [41.90; 43.66] | 42.52 *** [41.63; 43.40] |
| *Notes:* Shown values are beta coefficients, with 95% confidence intervals in brackets. Standard errors are clustered at the household level.  Significance levels: < 0.05*, < 0.01**, < 0.001***.  Abbreviations: ref. = reference category; TCALE = total cumulative adverse life event score; SOCALE = self-oriented cumulative adverse life event score; OOCALE = other-oriented cumulative adverse life event score; UK = United Kingdom. | | | | |

**Sensitivity analysis 2:**

| Table A5: Descriptive distribution of accumulation scores, stratified by gender. | | | |
| --- | --- | --- | --- |
|  | Men  (n = 2232) | Women  (n = 2832) | p-value^a^ |
| **Accumulation scores** |  |  |  |
| TCALE | 263.46 (150.05) | 249.90 (137.76) | 0.001 |
| SOCALE | 150.81 (102.89) | 141.19 (101.79) | 0.001 |
| OOCALE | 112.65 (87.10) | 108.71 (78.08) | 0.090 |
| *Notes:* Mean values (standard deviation) are presented.  ^a^ p-values indicating gender differences from two-tailed t-tests.  Abbreviations: TCALE = total cumulative adverse life event score; SOCALE = self-oriented cumulative adverse life event score; OOCALE = other-oriented cumulative adverse life event score. | | | |

| Table A6: Logistic regression models predicting depressive symptomatology. | | | | |
| --- | --- | --- | --- | --- |
|  | **Model 1** | **Model 2** | **Model 3** | **Model 4** |
| TCALE | 1.00 *** [1.00; 1.00] | - | 1.00 *** [1.00; 1.00] | - |
| SOCALE | - | 1.00 *** [1.00; 1.01] | - | 1.00 *** [1.00; 1.01] |
| OOCALE | - | 1.00 *** [1.00; 1.00] | - | 1.00 ** [1.00; 1.00] |
| TCALE x Gender | - | - | 1.00 [1.00; 1.00] | - |
| SOCALE x Gender | - | - | - | 1.00 [1.00; 1.00] |
| OOCALE x Gender | - | - | - | 1.00 [1.00; 1.00] |
| Gender: Men (ref.) | - | - | - | - |
| Women | 1.65 *** [1.36; 1.99] | 1.65 *** [1.36; 1.99] | 1.17 [0.76; 1.78] | 1.21 [0.79; 1.84] |
| Age: ≤ 59 years (ref.) | - | - | - | - |
| 60-69 years | 0.87 [0.70; 1.09] | 0.84 [0.67; 1.05] | 0.87 [0.69; 1.09] | 0.84 [0.67; 1.05] |
| 70-79 years | 0.74 * [0.58; 0.95] | 0.73 * [0.57; 0.93] | 0.75 * [0.58; 0.95] | 0.73 * [0.57; 0.94] |
| 80+ years | 0.88 [0.63; 1.22] | 0.90 [0.65; 1.26] | 0.88 [0.63; 1.23] | 0.91 [0.65; 1.26] |
| Birthplace: UK (ref.) | - | - | - | - |
| Outside of UK | 1.60 ** [1.12; 2.28] | 1.64 ** [1.15; 2.34] | 1.61 ** [1.13; 2.30] | 1.65 ** [1.16; 2.35] |
| Highest education: No qualification (ref.) | - | - | - | - |
| Low education | 0.60 *** [0.48; 0.75] | 0.60 *** [0.48; 0.75] | 0.60 *** [0.48; 0.75] | 0.60 *** [0.48; 0.75] |
| Medium education | 0.48 *** [0.38; 0.62] | 0.49 *** [0.38; 0.63] | 0.48 *** [0.38; 0.62] | 0.49 *** [0.38; 0.62] |
| High education | 0.27 *** [0.20; 0.38] | 0.27 *** [0.20; 0.38] | 0.27 *** [0.20; 0.38] | 0.27 *** [0.20; 0.38] |
| Intercept | 0.06 *** [0.04; 0.08] | 0.06 *** [0.04; 0.08] | 0.07 *** [0.05; 0.11] | 0.07 *** [0.05; 0.11] |
| *Notes:* Shown values are odds ratios, with 95% confidence intervals in brackets. Standard errors are clustered at the household level.  Significance levels: < 0.05*, < 0.01**, < 0.001***.  Abbreviations: ref. = reference category; TCALE = total cumulative adverse life event score; SOCALE = self-oriented cumulative adverse life event score; OOCALE = other-oriented cumulative adverse life event score; UK = United Kingdom. | | | | |

| Table A7: Linear regression models predicting life satisfaction. | | | | |
| --- | --- | --- | --- | --- |
|  | **Model 1** | **Model 2** | **Model 3** | **Model 4** |
| TCALE | -0.01 *** [-0.01; -0.01] | - | -0.01 *** [-0.01; -0.01] | - |
| SOCALE | - | -0.01 *** [-0.02; -0.01] | - | -0.01 *** [-0.02; -0.01] |
| OOCALE | - | -0.01 *** [-0.01; -0.00] | - | -0.00 * [-0.01; -0.00] |
| TCALE x Gender | - | - | -0.00 ** [-0.01; -0.00] | - |
| SOCALE x Gender | - | - | - | -0.00 [-0.01; 0.00] |
| OOCALE x Gender | - | - | - | -0.01 * [-0.01; -0.00] |
| Gender: Men (ref.) | - | - | - | - |
| Women | -0.32 * [-0.65; -0.00] | -0.34 * [-0.66; -0.01] | 0.60 [-0.05; 1.26] | 0.52 [-0.13; 1.18] |
| Age: ≤ 59 years (ref.) | - | - | - | - |
| 60-69 years | 0.57 * [0.13; 1.02] | 0.72 ** [0.27; 1.17] | 0.57 * [0.13; 1.02] | 0.72 ** [0.27; 1.17] |
| 70-79 years | 1.60 *** [1.14; 2.06] | 1.71 *** [1.25; 2.18] | 1.59 *** [1.13; 2.05] | 1.72 *** [1.25; 2.18] |
| 80+ years | 1.37 *** [0.72; 2.03] | 1.29 *** [0.64; 1.95] | 1.36 *** [0.70; 2.02] | 1.29 *** [0.64; 1.95] |
| Birthplace: UK (ref.) | - | - | - | - |
| Outside of UK | 0.23 [-0.52; 0.98] | 0.14 [-0.60; 0.89] | 0.21 [-0.54; 0.96] | 0.13 [-0.62; 0.87] |
| Highest education: No qualification (ref.) | - | - | - | - |
| Low education | -0.20 [-0.66; 0.26] | -0.22 [-0.68; 0.24] | -0.21 [-0.67; 0.25] | -0.23 [-0.69; 0.23] |
| Medium education | 0.34 [-0.17; 0.85] | 0.28 [-0.23; 0.79] | 0.34 [-0.17; 0.85] | 0.27 [-0.24; 0.78] |
| High education | 0.82 ** [0.27; 1.37] | 0.78 ** [0.24; 1.33] | 0.83 ** [0.29; 1.38] | 0.78 ** [0.24; 1.33] |
| Intercept | 22.39 *** [21.77; 23.00] | 22.30 *** [21.69; 22.92] | 21.90 *** [21.21; 22.58] | 21.87 *** [21.19; 22.55] |
| *Notes:* Shown values are beta coefficients, with 95% confidence intervals in brackets. Standard errors are clustered at the household level.  Significance levels: < 0.05*, < 0.01**, < 0.001***.  Abbreviations: ref. = reference category; TCALE = total cumulative adverse life event score; SOCALE = self-oriented cumulative adverse life event score; OOCALE = other-oriented cumulative adverse life event score; UK = United Kingdom. | | | | |

| Table A8: Linear regression models predicting quality of life. | | | | |
| --- | --- | --- | --- | --- |
|  | **Model 1** | **Model 2** | **Model 3** | **Model 4** |
| TCALE | -0.01 *** [-0.02; -0.01] | - | -0.01 *** [-0.01; -0.01] | - |
| SOCALE | - | -0.02 *** [-0.02; -0.02] | - | -0.02 *** [-0.02; -0.01] |
| OOCALE | - | -0.01 *** [-0.01; -0.00] | - | -0.00 [-0.01; 0.00] |
| TCALE x Gender | - | - | -0.00 * [-0.01; -0.00] | - |
| SOCALE x Gender | - | - | - | -0.00 [-0.01; 0.00] |
| OOCALE x Gender | - | - | - | -0.00 [-0.01; 0.00] |
| Gender: Men (ref.) | - | - | - | - |
| Women | 0.60 ** [0.18; 1.02] | 0.58 ** [0.16; 1.00] | 1.48 *** [0.60; 2.37] | 1.34 ** [0.46; 2.22] |
| Age: ≤ 59 years (ref.) | - | - | - | - |
| 60-69 years | 0.54 [-0.02; 1.11] | 0.79 ** [0.22; 1.36] | 0.55 [-0.02; 1.12] | 0.79 ** [0.22; 1.36] |
| 70-79 years | 0.23 [-0.39; 0.84] | 0.41 [-0.21; 1.03] | 0.22 [-0.40; 0.84] | 0.42 [-0.20; 1.04] |
| 80+ years | -2.03 *** [-2.92; -1.14] | -2.16 *** [-3.05; -1.28] | -2.05 *** [-2.93; -1.16] | -2.16 *** [-3.05; -1.28] |
| Birthplace: UK (ref.) | - | - | - | - |
| Outside of UK | -0.60 [-1.57; 0.37] | -0.75 [-1.71; 0.21] | -0.62 [-1.59; 0.35] | -0.76 [-1.73; 0.20] |
| Highest education: No qualification (ref.) | - | - | - | - |
| Low education | 1.40 *** [0.77; 2.02] | 1.37 *** [0.75; 2.00] | 1.39 *** [0.77; 2.02] | 1.36 *** [0.74; 1.99] |
| Medium education | 2.65 *** [1.97; 3.33] | 2.56 *** [1.88; 3.24] | 2.65 *** [1.97; 3.33] | 2.55 *** [1.87; 3.23] |
| High education | 3.80 *** [3.09; 4.51] | 3.73 *** [3.02; 4.45] | 3.81 *** [3.10; 4.52] | 3.74 *** [3.02; 4.45] |
| Intercept | 42.99 *** [42.18; 43.80] | 42.86 *** [42.05; 43.66] | 42.52 *** [41.60; 43.44] | 42.47 *** [41.56; 43.38] |
| *Notes:* Shown values are beta coefficients, with 95% confidence intervals in brackets. Standard errors are clustered at the household level.  Significance levels: < 0.05*, < 0.01**, < 0.001***.  Abbreviations: ref. = reference category; TCALE = total cumulative adverse life event score; SOCALE = self-oriented cumulative adverse life event score; OOCALE = other-oriented cumulative adverse life event score; UK = United Kingdom. | | | | |

**Sensitivity analysis 3:**

| Table A9: Descriptive distribution of experienced adverse life events and accumulation scores, stratified by gender. | | | |
| --- | --- | --- | --- |
|  | Men  (n = 1817) | Women  (n = 2366) | p-value^b^ |
| **Self-oriented adverse life events^a^** |  |  |  |
| Divorce | 380 (20.9) | 548 (23.2) | 0.090 |
| Separation from a long-term or/and cohabiting partner | 328 (18.1) | 524 (22.1) | 0.001 |
| Diagnosis of a serious physical disease | 989 (54.4) | 1392 (58.8) | 0.005 |
| Job loss due to company closing | 464 (25.5) | 427 (18.0) | <0.001 |
| Experienced a natural disaster | 240 (13.2) | 205 (8.7) | <0.001 |
| Suffered a life-threatening illness or serious accident | 567 (31.2) | 519 (21.9) | <0.001 |
| Victim of a serious physical attack or assault | 125 (6.9) | 101 (4.3) | <0.001 |
| Victim of sexual assault | 51 (2.8) | 208 (8.8) | <0.001 |
| Fired a weapon or came under fire during combat | 192 (10.6) | 14 (0.6) | <0.001 |
| Experienced severe financial hardship | 313 (17.2) | 477 (20.2) | 0.018 |
| Physically abused by parents before age 16 | 61 (3.4) | 84 (3.6) | 0.800 |
| Experienced evacuation during war | 189 (10.4) | 178 (7.5) | <0.001 |
| **Other-oriented adverse life events^a^** |  |  |  |
| Death of long-term partner/spouse | 181 (10.0) | 481 (20.3) | <0.001 |
| Death of child | 100 (5.5) | 147 (6.2) | 0.369 |
| Death of father | 220 (12.1) | 285 (12.0) | 0.989 |
| Death of mother | 205 (11.3) | 219 (9.3) | 0.036 |
| Parental divorce before age 16 | 88 (4.8) | 118 (5.0) | 0.887 |
| Experienced violence between parents before age 16 | 322 (17.7) | 538 (22.7) | <0.001 |
| Witnessed serious injury or death in war or military action | 178 (9.8) | 32 (1.4) | <0.001 |
| Witnessed a fatal or violent accident (not war) | 415 (22.8) | 182 (7.7) | <0.001 |
| Lost a very close friend or relative in war or military service | 222 (12.2) | 177 (7.5) | <0.001 |
| Close friend or relative was at risk of death or died due to illness or serious accident | 1178 (64.8) | 1579 (66.7) | 0.209 |
| **Accumulation scores** |  |  |  |
| TCALE | 3.86 (2.21) | 3.57 (1.98) | <0.001 |
| SOCALE | 2.15 (1.50) | 1.98 (1.46) | <0.001 |
| OOCALE | 1.71 (1.26) | 1.59 (1.10) | 0.001 |
| *Notes:* Absolute numbers (percentages) are presented for individual adverse life events; mean values (standard deviation) are presented for accumulation scores.  ^a^ For single adverse life events, only participants who reported experiencing the event are shown. Non-experienced cases (coded as "No") are not presented.  ^b^ p-values indicating gender differences from chi-square tests for single adverse life events or two-tailed t-tests for accumulation scores.  Abbreviations: TCALE = total cumulative adverse life event score; SOCALE = self-oriented cumulative adverse life event score; OOCALE = other-oriented cumulative adverse life event score. | | | |

| Table A10: General characteristics of the study population stratified by gender and overall. | | | |
| --- | --- | --- | --- |
|  | Men  (n = 1817) | Women  (n = 2366) | Overall  (n = 4183) |
| Depressive symptomatology |  |  |  |
| No | 1677 (92.3) | 2053 (86.8) | 3730 (89.2) |
| Yes | 140 (7.7) | 313 (13.2) | 453 (10.8) |
| Life satisfaction score |  |  |  |
| Mean (SD) | 20.4 (6.3) | 20.0 (6.6) | 20.2 (6.5) |
| Quality of life score (CASP-19) |  |  |  |
| Mean (SD) | 41.7 (8.3) | 42.0 (8.5) | 41.9 (8.4) |
| Age |  |  |  |
| ≤ 59 years | 665 (36.6) | 971 (41.0) | 1636 (39.1) |
| 60-69 years | 618 (34.0) | 706 (29.8) | 1324 (31.7) |
| 70-79 years | 391 (21.5) | 504 (21.3) | 895 (21.4) |
| 80+ years | 143 (7.9) | 185 (7.8) | 328 (7.8) |
| Birthplace |  |  |  |
| UK | 1721 (94.7) | 2229 (94.2) | 3950 (94.4) |
| Outside of UK | 96 (5.3) | 137 (5.8) | 233 (5.6) |
| Highest education |  |  |  |
| No qualification | 301 (16.6) | 551 (23.3) | 852 (20.4) |
| Low education | 487 (26.8) | 871 (36.8) | 1358 (32.5) |
| Middle education | 532 (29.3) | 525 (22.2) | 1057 (25.3) |
| High education | 497 (27.4) | 419 (17.7) | 916 (21.9) |
| HH-equivalent income |  |  |  |
| Low | 403 (22.2) | 730 (30.9) | 1133 (27.1) |
| Medium | 609 (33.5) | 760 (32.1) | 1369 (32.7) |
| High | 805 (44.3) | 876 (37.0) | 1681 (40.2) |
| Labour force status |  |  |  |
| In labour force | 802 (44.1) | 908 (38.4) | 1710 (40.9) |
| Unemployed | 115 (6.3) | 424 (17.9) | 539 (12.9) |
| Retired | 900 (49.5) | 1034 (43.7) | 1934 (46.2) |
| Partnership status |  |  |  |
| Married | 1414 (77.8) | 1524 (64.4) | 2938 (70.2) |
| Cohabiting | 104 (5.7) | 112 (4.7) | 216 (5.2) |
| Neither | 299 (16.5) | 730 (30.9) | 1029 (24.6) |
| Num. HH children |  |  |  |
| Mean (SD) | 0.36 (0.7) | 0.34 (0.7) | 0.35 (0.7) |
| Social participation |  |  |  |
| Mean (SD) | 1.5 (1.3) | 1.6 (1.4) | 1.6 (1.4) |
| Social network size |  |  |  |
| Mean (SD) | 7.4 (4.4) | 8.0 (4.0) | 7.7 (4.2) |
| *Notes:* Absolute numbers (percentages) are presented for nominal and ordinal variables; mean values (standard deviation) are presented for continuous variables.  Abbreviations: SD = standard deviation; CASP-19 = Control, Autonomy, Self-realization, and Pleasure 19; UK = United Kingdom, HH = household. | | | |

| Table A11: Logistic regression models predicting depressive symptomatology. | | | | |
| --- | --- | --- | --- | --- |
|  | **Model 1** | **Model 2** | **Model 3** | **Model 4** |
| TCALE | 1.27 *** [1.21; 1.33] | - | 1.22 *** [1.13; 1.31] | - |
| SOCALE | - | 1.31 *** [1.23; 1.40] | - | 1.23 *** [1.10; 1.38] |
| OOCALE | - | 1.21 *** [1.11; 1.32] | - | 1.20 ** [1.06; 1.36] |
| TCALE x Gender | - | - | 1.07 [0.98; 1.18] | - |
| SOCALE x Gender | - | - | - | 1.10 [0.96; 1.27] |
| OOCALE x Gender | - | - | - | 1.02 [0.86; 1.21] |
| Gender: Men (ref.) | - | - | - | - |
| Women | 1.62 *** [1.28; 2.05] | 1.62 *** [1.28; 2.04] | 1.17 [0.71; 1.94] | 1.20 [0.73; 2.00] |
| Age: ≤ 59 years (ref.) | - | - | - | - |
| 60-69 years | 0.66 ** [0.48; 0.90] | 0.65 ** [0.47; 0.89] | 0.66 ** [0.48; 0.90] | 0.65 ** [0.47; 0.89] |
| 70-79 years | 0.56 ** [0.39; 0.82] | 0.56 ** [0.39; 0.81] | 0.57 ** [0.39; 0.82] | 0.57 ** [0.39; 0.83] |
| 80+ years | 0.53 ** [0.33; 0.84] | 0.54 ** [0.34; 0.86] | 0.53 ** [0.33; 0.85] | 0.55 * [0.34; 0.88] |
| Birthplace: UK (ref.) | - | - | - | - |
| Outside of UK | 1.75 ** [1.17; 2.62] | 1.78 ** [1.19; 2.67] | 1.76 ** [1.18; 2.64] | 1.79 ** [1.20; 2.69] |
| Highest education: No qualification (ref.) | - | - | - | - |
| Low education | 0.71 * [0.55; 0.93] | 0.71 * [0.55; 0.93] | 0.71 * [0.54; 0.93] | 0.71 * [0.54; 0.93] |
| Medium education | 0.68 * [0.50; 0.93] | 0.69 * [0.50; 0.93] | 0.68 * [0.50; 0.92] | 0.68 * [0.50; 0.92] |
| High education | 0.50 *** [0.33; 0.75] | 0.49 *** [0.33; 0.74] | 0.49 *** [0.33; 0.74] | 0.49 *** [0.33; 0.74] |
| HH-equivalent income: Low (ref.) | - | - | - | - |
| Medium | 0.85 [0.66; 1.10] | 0.86 [0.66; 1.11] | 0.85 [0.66; 1.10] | 0.86 [0.66; 1.11] |
| High | 0.50 *** [0.36; 0.70] | 0.50 *** [0.36; 0.70] | 0.50 *** [0.36; 0.70] | 0.50 *** [0.36; 0.70] |
| Labour force status: In labour force (ref.) | - | - | - | - |
| Unemployed | 2.15 *** [1.57; 2.95] | 2.13 *** [1.55; 2.93] | 2.18 *** [1.58; 2.99] | 2.16 *** [1.56; 2.97] |
| Retired | 1.38 [0.99; 1.93] | 1.37 [0.98; 1.92] | 1.38 [0.99; 1.93] | 1.37 [0.98; 1.91] |
| Partnership status: Married (ref.) | - | - | - | - |
| Cohabiting | 0.81 [0.45; 1.45] | 0.82 [0.46; 1.46] | 0.81 [0.45; 1.45] | 0.81 [0.45; 1.47] |
| Neither | 1.98 *** [1.56; 2.53] | 2.00 *** [1.57; 2.54] | 1.95 *** [1.53; 2.49] | 1.97 *** [1.55; 2.50] |
| Num. HH children | 1.03 [0.88; 1.21] | 1.04 [0.89; 1.22] | 1.03 [0.88; 1.21] | 1.04 [0.89; 1.22] |
| Social participation | 0.85 *** [0.78; 0.93] | 0.85 *** [0.78; 0.93] | 0.85 *** [0.78; 0.93] | 0.85 *** [0.78; 0.93] |
| Social network size | 0.94 *** [0.91; 0.97] | 0.94 *** [0.91; 0.97] | 0.94 *** [0.91; 0.97] | 0.94 *** [0.91; 0.97] |
| Intercept | 0.09 *** [0.06; 0.15] | 0.09 *** [0.06; 0.15] | 0.11 *** [0.07; 0.19] | 0.11 *** [0.07; 0.19] |
| *Notes:* Shown values are odds ratios, with 95% confidence intervals in brackets. Standard errors are clustered at the household level.  Significance levels: < 0.05*, < 0.01**, < 0.001***.  Abbreviations: ref. = reference category; TCALE = total cumulative adverse life event score; SOCALE = self-oriented cumulative adverse life event score; OOCALE = other-oriented cumulative adverse life event score; UK = United Kingdom. | | | | |

| Table A12: Linear regression models predicting life satisfaction. | | | | |
| --- | --- | --- | --- | --- |
|  | **Model 1** | **Model 2** | **Model 3** | **Model 4** |
| TCALE | -0.60 *** [-0.69; -0.50] | - | -0.52 *** [-0.65; -0.39] | - |
| SOCALE | - | -0.79 *** [-0.93; -0.66] | - | -0.72 *** [-0.91; -0.52] |
| OOCALE | - | -0.30 *** [-0.47; -0.13] | - | -0.26 * [-0.49; -0.03] |
| TCALE x Gender | - | - | -0.15 [-0.33; 0.03] | - |
| SOCALE x Gender | - | - | - | -0.14 [-0.40; 0.11] |
| OOCALE x Gender | - | - | - | -0.09 [-0.41; 0.22] |
| Gender: Men (ref.) | - | - | - | - |
| Women | 0.05 [-0.31; 0.41] | 0.06 [-0.31; 0.42] | 0.60 [-0.11; 1.31] | 0.50 [-0.21; 1.21] |
| Age: ≤ 59 years (ref.) | - | - | - | - |
| 60-69 years | 0.32 [-0.23; 0.87] | 0.42 [-0.13; 0.97] | 0.32 [-0.23; 0.87] | 0.42 [-0.13; 0.97] |
| 70-79 years | 1.23 *** [0.56; 1.91] | 1.31 *** [0.64; 1.98] | 1.21 *** [0.54; 1.89] | 1.29 *** [0.62; 1.96] |
| 80+ years | 1.79 *** [0.90; 2.69] | 1.72 *** [0.83; 2.62] | 1.77 *** [0.87; 2.66] | 1.70 *** [0.80; 2.59] |
| Birthplace: UK (ref.) | - | - | - | - |
| Outside of UK | 0.32 [-0.43; 1.06] | 0.22 [-0.52; 0.97] | 0.31 [-0.44; 1.05] | 0.22 [-0.52; 0.96] |
| Highest education: No qualification (ref.) | - | - | - | - |
| Low education | -0.70 ** [-1.22; -0.18] | -0.70 ** [-1.22; -0.18] | -0.69 ** [-1.21; -0.17] | -0.69 ** [-1.22; -0.17] |
| Medium education | -0.81 ** [-1.41; -0.22] | -0.84 ** [-1.43; -0.25] | -0.80 ** [-1.39; -0.20] | -0.82 ** [-1.42; -0.23] |
| High education | -0.80 * [-1.46; -0.13] | -0.79 * [-1.45; -0.13] | -0.78 * [-1.44; -0.11] | -0.77 * [-1.43; -0.11] |
| HH-equivalent income: Low (ref.) | - | - | - | - |
| Medium | 0.68 * [0.14; 1.21] | 0.66 * [0.13; 1.19] | 0.68 * [0.14; 1.21] | 0.66 * [0.13; 1.19] |
| High | 1.46 *** [0.89; 2.04] | 1.40 *** [0.83; 1.97] | 1.45 *** [0.88; 2.03] | 1.40 *** [0.83; 1.97] |
| Labour force status: In labour force (ref.) | - | - | - | - |
| Unemployed | -1.60 *** [-2.29; -0.91] | -1.55 *** [-2.24; -0.86] | -1.61 *** [-2.30; -0.92] | -1.57 *** [-2.26; -0.88] |
| Retired | 0.65 * [0.10; 1.20] | 0.66 * [0.11; 1.21] | 0.65 * [0.09; 1.20] | 0.66 * [0.10; 1.21] |
| Partnership status: Married (ref.) | - | - | - | - |
| Cohabiting | 0.21 [-0.68; 1.10] | 0.22 [-0.67; 1.11] | 0.21 [-0.68; 1.11] | 0.22 [-0.67; 1.11] |
| Neither | -2.98 *** [-3.50; -2.46] | -3.03 *** [-3.55; -2.51] | -2.94 *** [-3.46; -2.42] | -3.00 *** [-3.52; -2.48] |
| Num. HH children | -0.32 * [-0.63; -0.00] | -0.37 * [-0.68; -0.05] | -0.31 * [-0.63; -0.00] | -0.37 * [-0.68; -0.05] |
| Social participation | 0.42 *** [0.28; 0.56] | 0.41 *** [0.27; 0.55] | 0.42 *** [0.28; 0.56] | 0.41 *** [0.27; 0.55] |
| Social network size | 0.26 *** [0.21; 0.30] | 0.25 *** [0.21; 0.30] | 0.25 *** [0.21; 0.30] | 0.25 *** [0.21; 0.30] |
| Intercept | 19.72 *** [18.87; 20.56] | 19.69 *** [18.85; 20.53] | 19.43 *** [18.52; 20.33] | 19.46 *** [18.55; 20.36] |
| *Notes:* Shown values are beta coefficients, with 95% confidence intervals in brackets. Standard errors are clustered at the household level.  Significance levels: < 0.05*, < 0.01**, < 0.001***.  Abbreviations: ref. = reference category; TCALE = total cumulative adverse life event score; SOCALE = self-oriented cumulative adverse life event score; OOCALE = other-oriented cumulative adverse life event score; UK = United Kingdom. | | | | |

| Table A13: Linear regression models predicting quality of life. | | | | |
| --- | --- | --- | --- | --- |
|  | **Model 1** | **Model 2** | **Model 3** | **Model 4** |
| TCALE | -0.82 *** [-0.95; -0.70] | - | -0.71 *** [-0.89; -0.53] | - |
| SOCALE | - | -1.10 *** [-1.28; -0.93] | - | -1.01 *** [-1.26; -0.76] |
| OOCALE | - | -0.40 *** [-0.63; -0.17] | - | -0.30 [-0.62; 0.02] |
| TCALE x Gender | - | - | -0.23 [-0.47; 0.02] | - |
| SOCALE x Gender | - | - | - | -0.17 [-0.50; 0.16] |
| OOCALE x Gender | - | - | - | -0.21 [-0.64; 0.21] |
| Gender: Men (ref.) | - | - | - | - |
| Women | 0.63 ** [0.16; 1.10] | 0.64 ** [0.17; 1.11] | 1.47 ** [0.52; 2.41] | 1.34 ** [0.39; 2.29] |
| Age: ≤ 59 years (ref.) | - | - | - | - |
| 60-69 years | 0.15 [-0.55; 0.85] | 0.30 [-0.40; 0.99] | 0.15 [-0.55; 0.85] | 0.29 [-0.41; 0.99] |
| 70-79 years | -0.36 [-1.24; 0.52] | -0.25 [-1.13; 0.63] | -0.39 [-1.27; 0.49] | -0.27 [-1.15; 0.61] |
| 80+ years | -2.19 *** [-3.38; -1.01] | -2.30 *** [-3.48; -1.11] | -2.23 *** [-3.42; -1.05] | -2.32 *** [-3.51; -1.14] |
| Birthplace: UK (ref.) | - | - | - | - |
| Outside of UK | -0.28 [-1.26; 0.70] | -0.41 [-1.38; 0.56] | -0.29 [-1.27; 0.69] | -0.42 [-1.39; 0.56] |
| Highest education: No qualification (ref.) | - | - | - | - |
| Low education | 0.64 [-0.07; 1.35] | 0.63 [-0.08; 1.34] | 0.65 [-0.06; 1.37] | 0.64 [-0.07; 1.35] |
| Medium education | 0.85 * [0.05; 1.65] | 0.81 * [0.02; 1.61] | 0.87 * [0.08; 1.67] | 0.83 * [0.04; 1.62] |
| High education | 1.06 * [0.19; 1.94] | 1.07 * [0.20; 1.94] | 1.09 * [0.22; 1.97] | 1.09 * [0.22; 1.97] |
| HH-equivalent income: Low (ref.) | - | - | - | - |
| Medium | 0.34 [-0.37; 1.05] | 0.31 [-0.39; 1.02] | 0.34 [-0.37; 1.05] | 0.31 [-0.40; 1.02] |
| High | 1.88 *** [1.14; 2.61] | 1.80 *** [1.07; 2.53] | 1.87 *** [1.13; 2.60] | 1.79 *** [1.06; 2.52] |
| Labour force status: In labour force (ref.) | - | - | - | - |
| Unemployed | -3.49 *** [-4.42; -2.56] | -3.43 *** [-4.36; -2.50] | -3.51 *** [-4.44; -2.58] | -3.45 *** [-4.38; -2.52] |
| Retired | -0.08 [-0.79; 0.63] | -0.07 [-0.78; 0.65] | -0.08 [-0.80; 0.63] | -0.07 [-0.79; 0.64] |
| Partnership status: Married (ref.) | - | - | - | - |
| Cohabiting | 1.60 ** [0.44; 2.75] | 1.61 ** [0.46; 2.75] | 1.60 ** [0.45; 2.76] | 1.61 ** [0.46; 2.76] |
| Neither | -1.08 ** [-1.74; -0.42] | -1.16 *** [-1.82; -0.50] | -1.02 ** [-1.69; -0.36] | -1.10 ** [-1.77; -0.44] |
| Num. HH children | -0.84 *** [-1.23; -0.45] | -0.92 *** [-1.31; -0.53] | -0.84 *** [-1.23; -0.45] | -0.91 *** [-1.30; -0.52] |
| Social participation | 0.87 *** [0.68; 1.05] | 0.85 *** [0.67; 1.03] | 0.87 *** [0.68; 1.05] | 0.85 *** [0.67; 1.03] |
| Social network size | 0.34 *** [0.28; 0.40] | 0.34 *** [0.28; 0.39] | 0.34 *** [0.28; 0.40] | 0.34 *** [0.28; 0.39] |
| Intercept | 40.24 *** [39.14; 41.34] | 40.20 *** [39.11; 41.30] | 39.81 *** [38.64; 40.97] | 39.85 *** [38.68; 41.01] |
| *Notes:* Shown values are beta coefficients, with 95% confidence intervals in brackets. Standard errors are clustered at the household level.  Significance levels: < 0.05*, < 0.01**, < 0.001***.  Abbreviations: ref. = reference category; TCALE = total cumulative adverse life event score; SOCALE = self-oriented cumulative adverse life event score; OOCALE = other-oriented cumulative adverse life event score; UK = United Kingdom. | | | | |

**Sensitivity analysis 4:**

| Table A14: General characteristics of the study population stratified by gender and overall. | | | |
| --- | --- | --- | --- |
|  | Men  (n = 2232) | Women  (n = 2832) | Overall  (n = 5064) |
| Depressive symptomatology |  |  |  |
| No | 1904 (85.3) | 2224 (78.5) | 4128 (81.5) |
| Yes | 328 (14.7) | 608 (21.5) | 936 (18.5) |
| Quality of life score (CASP-12) |  |  |  |
| Mean (SD) | 26.31 (5.63) | 26.47 (5.69) | 26.40 (5.67) |
| *Notes:* Absolute numbers (percentages) are presented for nominal and ordinal variables; mean values (standard deviation) are presented for continuous variables.  Abbreviations: SD = standard deviation; CASP-12 = Control, Autonomy, Self-realization, and Pleasure 12. | | | |

| Table A15: Logistic regression models predicting depressive symptomatology. | | | | |
| --- | --- | --- | --- | --- |
|  | **Model 1** | **Model 2** | **Model 3** | **Model 4** |
| TCALE | 1.27 *** [1.23; 1.31] | - | 1.22 *** [1.16; 1.28] | - |
| SOCALE | - | 1.33 *** [1.27; 1.40] | - | 1.31 *** [1.21; 1.43] |
| OOCALE | - | 1.18 *** [1.10; 1.25] | - | 1.10 * [1.01; 1.21] |
| TCALE x Gender | - | - | 1.07 * [1.00; 1.15] | - |
| SOCALE x Gender | - | - | - | 1.03 [0.93; 1.13] |
| OOCALE x Gender | - | - | - | 1.13 [1.00; 1.27] |
| Gender: Men (ref.) | - | - | - | - |
| Women | 1.59 *** [1.36; 1.85] | 1.58 *** [1.36; 1.84] | 1.16 [0.83; 1.61] | 1.19 [0.86; 1.66] |
| Age: ≤ 59 years (ref.) | - | - | - | - |
| 60-69 years | 0.89 [0.74; 1.08] | 0.86 [0.71; 1.04] | 0.89 [0.74; 1.07] | 0.86 [0.71; 1.04] |
| 70-79 years | 0.84 [0.69; 1.03] | 0.82 [0.67; 1.01] | 0.84 [0.69; 1.03] | 0.82 [0.67; 1.01] |
| 80+ years | 1.06 [0.81; 1.40] | 1.09 [0.82; 1.43] | 1.07 [0.81; 1.41] | 1.09 [0.83; 1.43] |
| Birthplace: UK (ref.) | - | - | - | - |
| Outside of UK | 1.39 * [1.02; 1.89] | 1.42 * [1.04; 1.93] | 1.40 * [1.03; 1.90] | 1.42 * [1.05; 1.94] |
| Highest education: No qualification (ref.) | - | - | - | - |
| Low education | 0.59 *** [0.49; 0.71] | 0.59 *** [0.49; 0.71] | 0.59 *** [0.49; 0.71] | 0.59 *** [0.49; 0.72] |
| Medium education | 0.49 *** [0.40; 0.60] | 0.49 *** [0.40; 0.61] | 0.49 *** [0.39; 0.60] | 0.50 *** [0.40; 0.61] |
| High education | 0.30 *** [0.23; 0.39] | 0.30 *** [0.23; 0.39] | 0.30 *** [0.23; 0.39] | 0.30 *** [0.23; 0.39] |
| Intercept | 0.12 *** [0.09; 0.16] | 0.12 *** [0.09; 0.16] | 0.14 *** [0.10; 0.20] | 0.14 *** [0.10; 0.20] |
| *Notes:* Shown values are odds ratios, with 95% confidence intervals in brackets. Standard errors are clustered at the household level.  Significance levels: < 0.05*, < 0.01**, < 0.001***.  Abbreviations: ref. = reference category; TCALE = total cumulative adverse life event score; SOCALE = self-oriented cumulative adverse life event score; OOCALE = other-oriented cumulative adverse life event score; UK = United Kingdom. | | | | |

| Table A16: Linear regression models predicting quality of life. | | | | |
| --- | --- | --- | --- | --- |
|  | **Model 1** | **Model 2** | **Model 3** | **Model 4** |
| TCALE | -0.59 *** [-0.67; -0.51] | - | -0.50 *** [-0.62; -0.39] | - |
| SOCALE | - | -0.79 *** [-0.89; -0.68] | - | -0.74 *** [-0.90; -0.58] |
| OOCALE | - | -0.30 *** [-0.44; -0.15] | - | -0.19 [-0.39; 0.01] |
| TCALE x Gender | - | - | -0.17 * [-0.32; -0.02] | - |
| SOCALE x Gender | - | - | - | -0.09 [-0.30; 0.12] |
| OOCALE x Gender | - | - | - | -0.22 [-0.49; 0.05] |
| Gender: Men (ref.) | - | - | - | - |
| Women | 0.30 * [0.01; 0.59] | 0.30 * [0.01; 0.59] | 0.94 ** [0.34; 1.53] | 0.85 ** [0.25; 1.44] |
| Age: ≤ 59 years (ref.) | - | - | - | - |
| 60-69 years | 0.48 * [0.10; 0.87] | 0.61 ** [0.22; 1.00] | 0.48 * [0.10; 0.87] | 0.61 ** [0.22; 0.99] |
| 70-79 years | 0.19 [-0.23; 0.61] | 0.30 [-0.12; 0.71] | 0.18 [-0.24; 0.60] | 0.29 [-0.13; 0.71] |
| 80+ years | -1.51 *** [-2.11; -0.90] | -1.57 *** [-2.17; -0.97] | -1.52 *** [-2.12; -0.92] | -1.58 *** [-2.18; -0.98] |
| Birthplace: UK (ref.) | - | - | - | - |
| Outside of UK | -0.33 [-0.97; 0.32] | -0.41 [-1.05; 0.23] | -0.34 [-0.99; 0.31] | -0.41 [-1.06; 0.23] |
| Highest education: No qualification (ref.) | - | - | - | - |
| Low education | 1.01 *** [0.58; 1.43] | 0.98 *** [0.56; 1.41] | 1.00 *** [0.58; 1.43] | 0.98 *** [0.56; 1.40] |
| Medium education | 1.77 *** [1.30; 2.23] | 1.70 *** [1.24; 2.17] | 1.77 *** [1.30; 2.23] | 1.70 *** [1.23; 2.16] |
| High education | 2.62 *** [2.14; 3.10] | 2.57 *** [2.08; 3.05] | 2.63 *** [2.15; 3.11] | 2.57 *** [2.09; 3.06] |
| Intercept | 27.08 *** [26.53; 27.62] | 26.98 *** [26.44; 27.53] | 26.74 *** [26.13; 27.36] | 26.71 *** [26.10; 27.32] |
| *Notes:* Shown values are beta coefficients, with 95% confidence intervals in brackets. Standard errors are clustered at the household level.  Significance levels: < 0.05*, < 0.01**, < 0.001***.  Abbreviations: ref. = reference category; TCALE = total cumulative adverse life event score; SOCALE = self-oriented cumulative adverse life event score; OOCALE = other-oriented cumulative adverse life event score; UK = United Kingdom. | | | | |
