# Supplementary material for: The accumulation of adverse life events across the life course and their association with mental health and well-being: exploring gender heterogeneity
Source: BMC Public Health. 2026 Oct 23;26:3054. doi: 10.1186/s12889-026-30076-7 (PMC13693152; doi:10.1186/s12889-026-30076-7)
Supplement: Supplementary file 6 [file 12889_2026_30076_MOESM6_ESM.docx]

**Additional File 6**

**Title: Additional descriptive information on accumulation scores and well-being outcomes.**

| Table A1. 25th percentile, median, and 75th percentile of the accumulation scores and well-being outcomes. | | | | | | | | | | | |  |
| --- | --- | --- | --- | --- | --- | --- | --- | --- | --- | --- | --- | --- |
|  | Men  (n = 2232) | | | Women  (n = 2832) | | | | Overall  (n = 5064) | | | |  |
|  | P25 | Median | P75 | | P25 | Median | P75 | | P25 | Median | P75 | |
| TCALE | 2 | 4 | 5 | | 2 | 3 | 5 | | 2 | 4 | 5 | |
| SOCALE | 1 | 2 | 3 | | 1 | 2 | 3 | | 1 | 2 | 4 | |
| OOCALE | 1 | 2 | 2 | | 1 | 1 | 2 | | 1 | 1 | 2 | |
| Life satisfaction score | 17 | 22 | 25 | | 16 | 22 | 25 | | 17 | 22 | 25 | |
| Quality of life score | 36 | 43 | 48 | | 37 | 43 | 48 | | 37 | 43 | 48 | |
| *Notes:* Values represent the 25th percentile (P25), median, and 75th percentile (P75).  Abbreviations: TCALE = total cumulative adverse life event score; SOCALE = self-oriented cumulative adverse life event score; OOCALE = other-oriented cumulative adverse life event score. | | | | | | | | | | | |  |
